# Phenotypic and genotypic divergence of plant–herbivore interactions along an urbanization gradient

**DOI:** 10.1111/eva.13376

**Published:** 2022-04-25

**Authors:** Jiao Qu, Dries Bonte, Martijn L. Vandegehuchte

**Affiliations:** ^1^ Lushan Botanical Garden Chinese Academy of Sciences Jiangxi China; ^2^ Terrestrial Ecology Unit Department of Biology Ghent University Ghent Belgium; ^3^ Department of Biology Norwegian University of Science and Technology Trondheim Norway

**Keywords:** *Arabidopsis thaliana*, evolution, fitness, plant–herbivore interactions, plasticity, urbanization

## Abstract

Urban environments provide challenging conditions for species survival, including increased temperatures, drought and pollution. Species can deal with these conditions through evolution across generations or the immediate expression of phenotypic plasticity. The resulting phenotypic changes are key to the performance of species and their interactions with other species in the community. We here document patterns of herbivory in *Arabidopsis thaliana* along a rural–urban gradient, and tested the genetic background and ecological consequences of traits related to herbivore resistance. Aphid densities increased with urbanization levels along the gradient while plant size did not change. Offspring of urban mothers, raised under common garden conditions, were larger and had a decreased trichome density and seed set but a higher caterpillar (*Pieris brassicae*) tolerance. In contrast, no urban evolution was detected for defences against aphids (*Myzus persicae*). Aphids reduced seed set more strongly in urban offspring, but this effect disappeared in second‐generation plants. In general, urban adaptations as expressed in size and caterpillar tolerance were found, but these adaptations were associated with smaller inflorescences. The maternal effect on the response of seed set to aphid feeding demonstrates the relevance of intergenerational plasticity as a direct ecological consequence of herbivory. Our study demonstrates that the urban environment interacts with the plant's genotype and the extended phenotype as determined by ecological interactions.

## 1 INTRODUCTION

Urbanization alters abiotic and biotic environments across time and space (McDonnell & Hahs, 2015), as a result of the fragmentation of natural habitat and increased pollution, temperature, impervious surface area and numbers of invasive species (Grimm et al., 2008; Niemelä et al., 2011). These environmental changes have greatly altered species’ population dynamics, community structure (Alberti, 2015) and species interaction networks (Delgado‐V & French, 2012; El‐Sabaawi, 2018; Martin & Bonier, 2018; Meyer et al., 2020). Some species rapidly adapt to urban environments, not only through phenotypic plasticity (Alberti et al., 2017), but also through local genetic adaptation (McDonnell & Hahs, 2015). Evolutionary changes have been demonstrated mostly for vertebrates, but rarely for invertebrates and plants (Johnson & Munshi‐South, 2017). These genetic changes cause trait shifts, which may subsequently affect interactions with other species (Irwin et al., 2014; Start et al., 2018), and therefore influence the entire species community.

Herbivory is a key ecosystem process as it governs biomass production and energy flows among trophic levels (Speight et al., 1999), with important effects on ecosystem functioning. Strong declines in arthropod biomass (Dahirel et al., 2019; Svenningsen et al., 2020) and diversity (Faeth et al., 2011) in response to urbanization seem to be a rule rather than an exception. Nevertheless, the impact of urbanization on herbivory is difficult to predict (El‐Sabaawi, 2018) and often guild‐specific (Kozlov et al., 2017; Moreira et al., 2018). For example, small, less mobile, sap‐sucking arthropods with short generation times (e.g. aphids and scale insects) generally increase in abundance with urbanization (Mcintyre, 2000; Raupp et al., 2009; Youngsteadt et al., 2015). Differential changes across trophic levels, such as increased sap‐sucking insect densities combined with decreased predatory arthropod abundance (Corcos et al., 2019; Rocha‐Filho et al., 2020), could disrupt food webs and promote outbreaks of insect pests in cities (Korányi et al., 2021). Abiotic and biotic factors associated with urbanization can be direct drivers of insect herbivory, by causing changes in insect abundance and diversity (Dale & Frank, 2018; Fenoglio et al., 2021). Besides, shifts in phenology, physiology and behaviour of either herbivores or plants may drive the evolution of their interactions in an urban context (Miles et al., 2019). Especially relevant from this perspective is the evolution of traits related to plant nutritional quality or structural and chemical defences (Kozlov et al., 2017; Moreira et al., 2018; Thompson et al., 2016) as they directly affect the presence of associated species, that is the extended phenotype of the plant (Dawkins, 1982). Overall, our understanding of such eco‐evolutionary dynamics in cities is based on a few species. Urbanization for instance altered selection on a chemical defence against herbivores, but these evolved changes were unrelated to variation in herbivory (Johnson et al., 2018; Thompson et al., 2016).

Traits that enable plants to cope with harsh abiotic conditions may also indirectly affect their capacity to defend themselves against herbivores. Plants from urban environments may, for instance, grow larger with fewer leaves and bolt earlier (Yakub & Tiffin, 2017). Earlier bolting is speculated to represent an adaptation to the hotter, drier urban microclimate and larger size to the altered competitive environment. Fewer leaves may also cause resource limitation to herbivore growth, thus reducing herbivory (Buckley et al., 2019). Structural defences against herbivores can also protect plants from abiotic pressures. For instance, leaf trichomes may protect plants from drought (Sletvold & Ågren, 2012) resulting from the urban heat island effect. Consequently, assessing changes in diverse growth and defence traits of plants along the urbanization gradient can shed light on urban adaptations.

Confirming evolutionary change in these traits requires assessing whether observed phenotypic divergence has a genetic basis (Donihue & Lambert, 2015). Common garden experiments achieve this by quantifying the relative extent to which the genetic and environmental component as well as their interaction contribute to trait change. While considering the effects of both abiotic and biotic factors on trait variation is indispensable for understanding potential adaptive responses of plants to urbanization, their relative importance has not been explored yet. This currently lacking information is crucial for urban planners to optimize plant performance and mitigate risks of pest outbreaks in urban green spaces today as well as under changing biotic and abiotic conditions brought on by climate change.

*Arabidopsis thaliana*can cope with herbivores via tolerance, including compensatory growth, or resistance, either morphological via trichomes or chemical via glucosinolates, which are unpalatable or even toxic to various insect herbivores (Mauricio, 1998). Different herbivore feeding guilds can elicit different signalling pathways resulting in specific phenotypic changes in *A*. *thaliana* (Davila Olivas et al., 2017). We surveyed invertebrate herbivores and several performance metrics of wild *Arabidopsis thaliana* along an urbanization gradient in Belgium. We further exposed multiple genetic lines from this urbanization gradient to a phloem‐sucking aphid, *Myzus persicae*, and a leaf‐chewing caterpillar, *Pieris brassicae*, under common garden conditions to investigate tolerance and resistance traits. Potential maternal effects were assessed by growing a selection of these lines for a second generation. In doing so, we tested whether (1) plant–insect interactions diverge phenotypically along the urbanization gradient, and (2) whether such a divergence stems from genetic variation in plant traits related to tolerance or resistance against herbivory. We hypothesize that (1) sap‐sucking arthropods increase, while other arthropod groups decrease, in abundance on *A*. *thaliana* towards the city center; (2) this has resulted in adaptive responses in *A*. *thaliana*, such as lowered resistance against leaf chewers in favour of increased resistance against sap suckers, either via evolution of these traits, increased phenotypic plasticity, or maternal effects.

## 2 MATERIALS AND METHODS

### 2.1 Study species

*Arabidopsis thaliana* (L.) Heynh. (Brassicaceae) is a cosmopolitan annual plant native to Western Eurasia. It is self‐fertilizing and wild populations are highly homozygous. *A*. *thaliana* has shown evolution of resistance and tolerance to natural herbivory (Weinig et al., 2003). It can adapt to local herbivore communities along a longitudinal gradient (Brachi et al., 2015), and also to heat and drought along a decreasing elevational gradient (Wolfe & Tonsor, 2014).

### 2.2 Field survey of plant‐herbivore interactions

In May 2017, when *A*. *thaliana* matured, a field survey was conducted within an area of approximately 8 by 8 km² in and around the city of Ghent in Belgium. This area is composed of highly urbanized, agricultural and semi‐natural areas (Figure S1). In total, 104 individual *A*. *thaliana* plants were sampled across these land‐use gradients, at least 50 m from each other.

Before harvesting plants, the location of each plant was recorded with a hand‐held GPS device (±5 m accuracy). As a relevant proxy for urbanization (Raupp et al., 2009; Rocha & Fellowes, 2018) we used percentage of built‐up cover, which was assessed in a geographic information system (GIS) as in Merckx et al. (2018). The responses of species to landscape alterations depend on their mobility and on the spatial scales at which ecological processes tend to act on species (Concepción et al., 2015; Piano et al., 2016). Therefore, changes in urbanization‐induced stresses along urbanization gradients at several spatial scales should be taken into account when assessing the response of organisms to urbanization. Built‐up cover was therefore quantified as a continuous variable for a nested set of spatial scales (50, 100, 200, 400, 800, 1600, 2400 and 3200 m radii, Figure S1) around each sample (Merckx et al., 2018).

For each plant, 11 plant traits were measured (see Appendix S1 for more information): number of conspecific plants within 1 m^2^ centred on the plant, plant height, dry shoot, root and total plant mass, water content of shoots and roots, ratio of root dry mass to total plant dry mass, number of matured fruits, mean fruit length, and total seed production (number of fruits multiplied with mean fruit length). Aphids were the main observed herbivores at the time of the survey, and were counted.

### 2.3 Assessment of plant tolerance and resistance against caterpillars and aphids in common garden experiments

#### 2.3.1 Plant material

Eighteen plants were selected from the 104 individuals, representing different levels of urbanization throughout the whole study area (Figure S1, Table S1), and their seeds were collected in the field. To exclude potential rare outcrossing at fine spatial scales, the 18 selected maternal plants had a minimum distance of 500 m between each other. Given the low outcrossing rates of *A*. *thaliana*, seeds from each of the 18 individual plants are highly related and hereafter referred to as families or ‘genotypes’ (sensu lato) or ‘lines’. Eighteen genotypes of the first generation were used in a caterpillar herbivory experiment, while the same 10 genotypes of the first and second generation were used for an aphid herbivory experiment (Table S1). Seeds to grow this second generation of plants were harvested from first‐generation plants grown in the greenhouse under the same conditions.

#### 2.3.2 Experiment (1): Caterpillar herbivory

Ten four‐week‐old plants of each genotype were randomly divided into even control and herbivory groups (see Appendix S2 for details on plant growing and insect rearing conditions). Each plant of the herbivory group received one pre‐weighed first‐instar larva of *P*. *brassicae*. Each plant was placed in a transparent plastic cage covered with netting (<0.35 mm mesh width) (Figure S2a). Herbivores fed for 4 days and were weighed daily to determine their growth rate, after which they were removed. Subsequently, all plants (*N* = 180) were harvested, split into above‐ and belowground parts and air‐dried between papers at room temperature.

Dry leaves were scanned (Canon CanoScan LiDE 100). Actual leaf area (ALA) was obtained through an automated program in ImageJ (US National Institutes of Health). For the herbivory group, we determined potential leaf area (PLA) by manually cloning the missing leaf parts in the image from the opposite side of the same leaf or from similarly sized leaves of the same plant to infer the initial total area (Unsicker et al., 2006). Afterwards, absolute leaf damage (ALD) was measured as PLA – ALA, and relative leaf damage (RLD) as ALD/PLA.

Leaf resistance traits to herbivores were also determined. For each plant, relative leaf resistance to herbivores (RLR) was calculated as 1 – RLD. If each caterpillar eats a similar absolute amount of leaf tissue over a given period of time, RLR as estimate of resistance may be biased because larger plants would then always appear to be relatively more resistant than smaller plants (Valverde et al., 2003). Therefore, we adopted the maximum absolute leaf damage (ALD_max_) across all plants as a reference (this is the no‐resistance scenario), and then scaled the absolute leaf area consumed to this reference damage to calculate scaled leaf resistance (SLR) as (ALD_max_ – ALD)/ALD_max_. The plants with most consumed leaf area have a SLR of 0, and plants with no leaf damage have a SLR of 1. On pressed and air‐dried plants, we counted the number of trichomes within a circle (6 mm diameter) in the central portion of the upper surface of one to ten fully developed leaves per plant under a dissection microscope. Leaf trichome density of each plant was calculated as the mean number of trichomes across these leaves as another measure of resistance to herbivores.

Finally, rosettes and roots were oven‐dried for at least 48 h at 50°C and weighed to the nearest 0.001 g. Plant tolerance to herbivores was assessed as the difference between the line mean (ML) of plants with herbivores (ML_P_) and of those without herbivores (ML_C_) divided by the latter ((ML_P_ – ML_C_)/ML_C_) in rosette biomass, root biomass, total biomass and actual leaf area (Leimu & Koricheva, 2006; Weinig et al., 2003). Caterpillar relative growth rate over the 4 days (Figure S3) was calculated as $\frac{\log(W_{i}/W_{0})}{t_{i}‐t_{0}}$ (*W_i_*, caterpillar mass at day *t*_i_; *W*_0_, initial mass of the caterpillar when introduced at *t*_0_).

#### 2.3.3 Experiment (2): Aphid herbivory

When some flowers had opened, plants of each genotype were paired according to the height of stalks (measured to the nearest 1 mm). Length of the longest rosette leaf was assessed using a digital calliper to the nearest 0.01 mm. One plant of each pair received a single first‐instar nymph of the aphid *M*. *persicae*. In total, 5 replicate plants of each genotype were exposed to the control and 5 to the aphid treatment (*N* = 100 per generation). Each plant was placed in a cage similar to the caterpillar cages but with different dimensions (Figure S2b). Aphids were counted in the morning every 3 or 4 days, but all plants were counted at the same frequency. Plants received the same amount of water on the same day. As plants grew, watering frequency was reduced from every 3 days to once a week. When plants senesced (no green tissue left), watering was stopped and plants were harvested after another week. Plant height was measured to the nearest 1 mm. The number of branches that produced fruits and the total number of fruits were recorded. Afterwards, ten randomly selected siliques at the bottom, middle and top positions of several branches were cut off, scanned, and their lengths calculated in ImageJ. Total seed number was estimated by multiplying the number of fruits with the mean length of the ten fruits. Finally, aboveground plant tissues were cut off at the soil surface, separated into stems and rosette leaves, and stored in envelopes. Stems and rosette leaves were oven‐dried for at least 48 h at 50°C and weighed to the nearest 0.001 g. Shoot biomass was taken as the sum of rosette leaf and stem biomass. Plant tolerance to aphids was assessed as the difference within each plant pair (DP) between the plant with aphids (DP_M_) and that without aphids (DP_C_) divided by the latter ((DP_M_ – DP_C_)/DP_C_) in rosette and stem dry mass and total seed number.

Stem growth since aphid introduction was calculated as the difference in plant height from the time of aphid introduction to the time of plant harvesting. The highest number of aphids counted over all days was taken as the population peak, that is the maximum population size that a plant can support. An exponential growth curve *N* = N_0_.e^rt^ was fitted through the aphid numbers from the day of aphid introduction until the day of population peak (Figure S4) and the growth constant r (Table S2) acted as a measure of aphid population growth rate.

### 2.4 Statistical analysis

All statistical analyses were performed using R 3.6.3 (R Development Core Team, 2018).

#### 2.4.1 Spatial scale of urbanization and general approach

For each response variable, we plotted *R* ^2^ values from (generalized) linear models and conditional *R* ^2^ values (*R* ^2^ *_c_*) from (generalized) linear mixed‐effect models against the eight scales at which urbanization was assessed (Figure S5–7). *R* ^2^ *_c_* values were estimated via Nakagawa and Schielzeth's *R* ^2^_GLMM_ (Johnson, 2014) with the function ‘r.squaredGLMM’ in the package ‘MuMIn’. Based on these plots, we decided to consider the scale of 200 metre for all models, as it was either one of scales with the highest *R* ^2^/*R* ^2^ *_c_* values or all values were very similar. Parametric bootstrap *p*‐values with 10,000 simulations for fixed effects were then estimated by the function ‘PBmodcomp’ (‘pbkrtest’ package) for linear models and by the method ‘PB’ in the function ‘mixed’ (‘afex’ package) for linear mixed models based on Type III sums of squares. *P*‐values of type‐III Chi^2^ Wald tests were calculated via function ‘Anova’ (‘car’ package) for negative binomial generalized linear (mixed‐effect) models. We provided raw *p*‐values and *p*‐values adjusted via the classical one‐stage method based on false discovery rates (Pike, 2011; Verhoeven et al., 2005). We applied False Discovery Rate corrections for effects of the same predictor variables or their interactions on different response variables, if the latter were measured on the exact same set of plants. For models fitting multiple slopes, a post‐hoc analysis of slopes was performed (function ‘emtrends’, ‘emmeans’ package).

#### 2.4.2 Field survey of plant–herbivore interactions

As many plant traits were correlated, we conducted a principal component analysis (PCA) on these standardized traits (mean = 0, SD = 1) using the ‘PCA’ function (‘FactoMineR’ package). We used linear models to assess urbanization effects on the first two axes (PC1 and PC2). The relationship between aphid abundance and urbanization was analysed using generalized linear models with a negative binomial distribution to account for overdispersion and a log link function (function ‘glm.nb’, ‘MASS’ package). To account for potential dependence of aphid abundance on resource availability, we included plant shoot dry mass as a covariate. We used spline correlograms (‘ncf’ package) to assess spatial autocorrelation of residuals of all models (Appendix S3).

#### 2.4.3 Experiment (1): caterpillar herbivory

As many of the six plant traits (rosette, root and total dry mass, root fraction of total mass, actual leaf area and leaf trichome density) were highly correlated, we performed a PCA as described above. The effects of caterpillars and urbanization of the mothers’ environment on the PC1 and PC2 scores were examined using separate linear mixed‐effect models (function ‘mixed’ in ‘afex’ package). Genotype was included as a random effect. The genotype‐by‐treatment interaction was dropped as random effect from all models because of singularity for almost all models because of a near‐zero variance component. Within the herbivory group, responses of resistance traits (absolute leaf damage, scaled leaf resistance, and caterpillar growth rate) to urbanization were analysed with linear mixed‐effect models with urbanization as fixed and genotype as random effect. Due to little variance explained by genotype, linear models were fitted for caterpillar growth rate. Caterpillar tolerance of actual leaf area and of rosette, root, and total mass were analysed in relation to urbanization with linear models. We also constructed linear mixed‐effect models with leaf trichome density as fixed and plant genotype as random effect and absolute leaf damage, scaled leaf resistance, or caterpillar growth rate as response variable. Finally, the relationship between actual leaf area and leaf trichome density was tested with a linear mixed‐effect model with actual leaf area as fixed and plant genotype as random effect.

#### 2.4.4 Experiment (2): aphid herbivory

As many of the ten plant traits (plant height, number of branches, length of the longest leaf, dry weights of rosette leaves, stems and shoots, numbers of fruits and seeds, mean fruit length, and plant height growth since aphid introduction) were highly correlated, a PCA (as described above) was performed. To test the effects of urbanization, plant generation, aphids and all their interactions on plant characteristics, linear mixed‐effect models were performed for the first two PCs. Genotype and the genotype‐by‐generation, genotype‐by‐treatment, genotype‐by‐generation‐by‐treatment and genotype‐by‐generation‐by‐pair interaction were initially included as random effects. As the genotype‐by‐generation interaction caused singularity for most models and explained little variance for others, it was removed. Two plants for which the aphids died shortly after introduction were treated as control plants. Within the aphid treatment, we tested the effects of urbanization, seed generation, and their interaction on aphid population growth rate via linear mixed‐effect models and on aphid abundance at population peak via negative binomial generalized linear mixed‐effect models. Genotype and the genotype‐by‐generation interaction were included as random effects. Dry shoot mass was included as a covariate in the models of aphid population peak. Urbanization and dry shoot mass were standardized in the models of aphid peak abundance. Effects of urbanization, seed generation and their interaction on aphid tolerance of dry rosette and stem mass and total seed number were analysed using linear mixed‐effect models. Genotype and the genotype‐by‐generation interaction were included as random effects. Because of singularity and near‐zero variance components, the interaction was removed for tolerance of total seed number and both random effects for tolerance of dry rosette leaf mass, for which linear models were fitted.

## 3 RESULTS

### 3.1 Field survey of plant‐herbivore interactions

**FIGURE 1:**
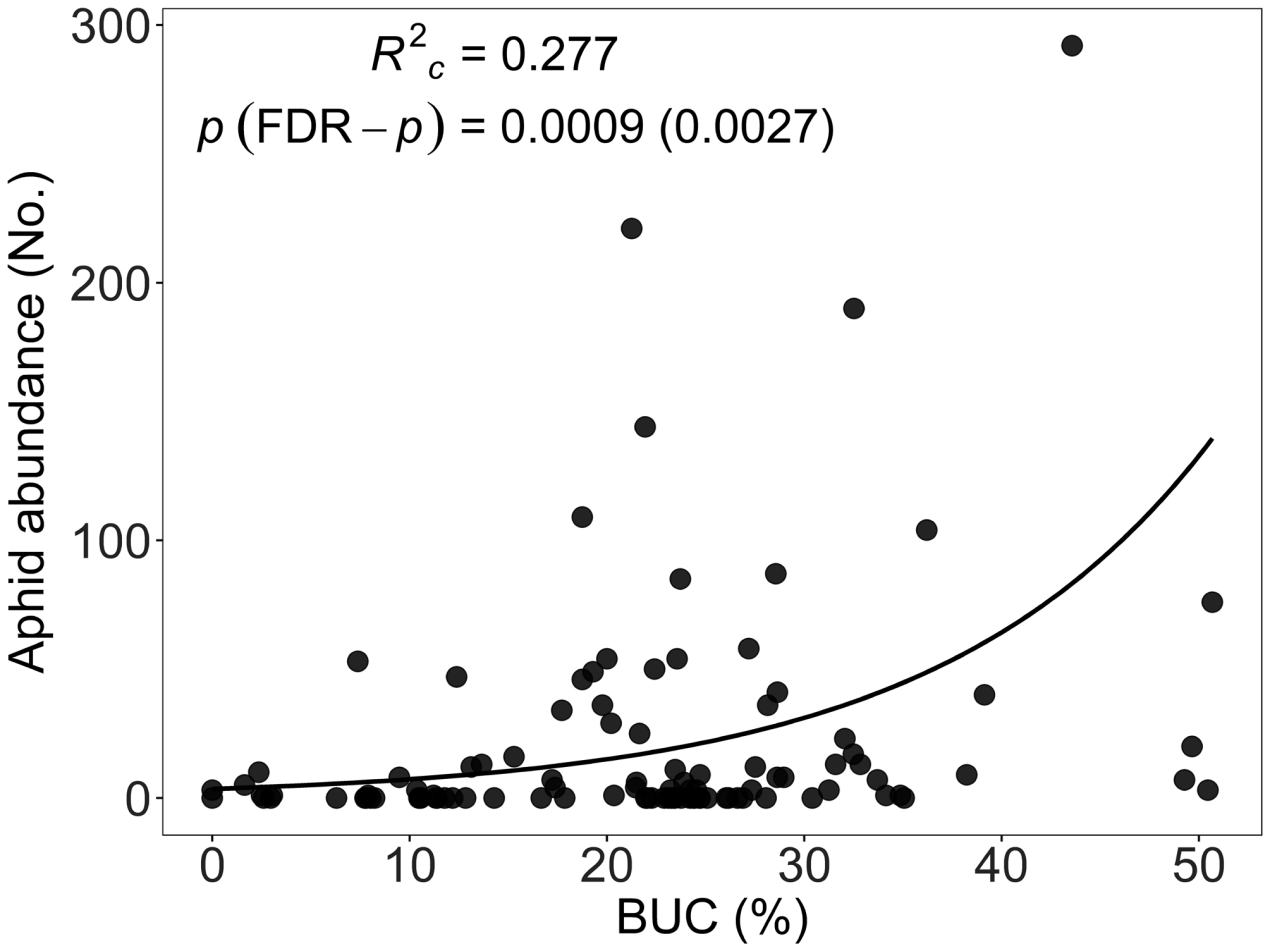
Relationship between aphid abundance on *Arabidopsis thaliana* and urbanization (% BUC: 200 m radius) in the field. A negative binomial generalized linear model was fitted with shoot dry mass as a covariate. The line is fitted for the mean value of shoot dry biomass. Model *R* ^2^, parametric bootstrap *p*‐value, and its FDR‐adjusted *p*‐value are provided. Estimates and standard errors of parameters are provided in Table S4

Higher values of the first plant trait PC represented increases in shoot biomass, whole plant biomass, total number of seeds and fruits, root biomass, plant height, and fruit length, and a decreased root biomass allocation (Table S3, Figure S8). Higher values of the second PC represented higher shoot moisture content, longer fruits and moister roots, and a lower conspecific plant density (Table S3, Figure S8). PC1 is thus mainly correlated with increases in plant size and fecundity and PC2 mainly with increased moisture and decreased local plant density. Urbanization levels varied widely, ranging from 0 to 55%. The 95% pointwise bootstrap confidence interval of the spline correlograms (Figure S9) included zero at all sample inter‐distances for the residuals of all tested models at all buffer radii. This indicates that the spatial autocorrelation of model residuals was never statistically significant, and therefore that spatial position of the samples did not need to be taken into account. In the field, neither PC1 (plant size and fecundity) nor PC2 (plant moisture content and conspecific plant density) was related to the proportion of built‐up area (Table S4). Absolute aphid abundance increased strongly with increasing built‐up cover (Figure 1) but the increase with plant dry shoot biomass was not significant (Table S4).

### 3.2 Experiment (1): caterpillar herbivory

**FIGURE 2:**
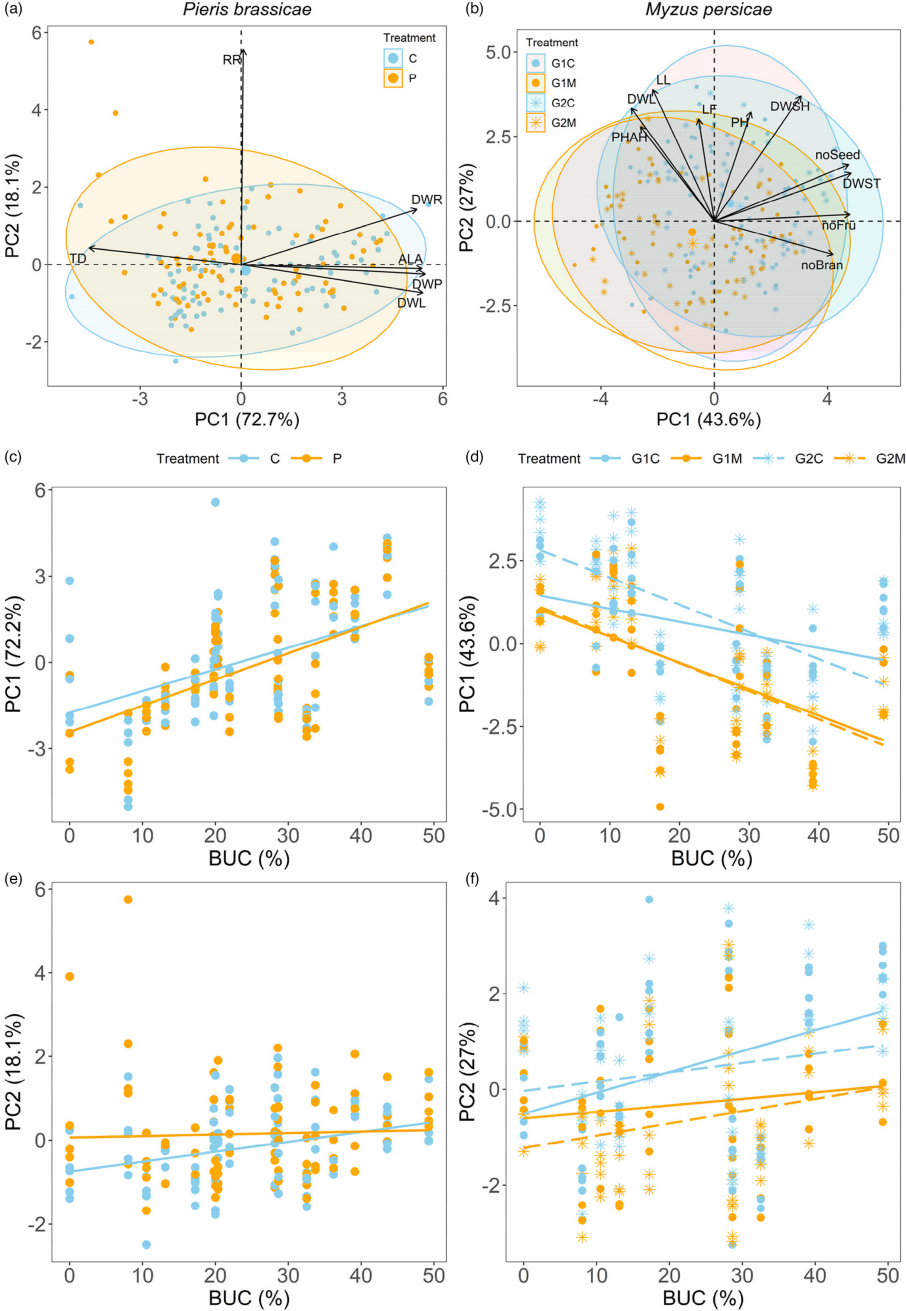
(a) PCA of standardized traits of *A*. *thaliana* grown from seeds of 18 mothers from locations varying in urbanization level. P: *P*. *brassicae* caterpillars, C: control. DHL: dry rosette leaf weight, DWR: dry root weight, DWP: dry total plant weight, RR: root to total plant mass ratio, ALA: actual total leaf area, TD: trichome density. (b) PCA on standardized traits of *A*. *thaliana* of the first (G1) and second (G2) generation from 10 of the 18 (grand)mothers. M: *M*. *persicae* aphids, C: control. PH: plant height at death, noBran: branch number, noFru: fruit number, LF: mean fruit length, DWL: dry rosette leaf weight, DWST: dry stem weight, DWSH: dry shoot weight, LL: longest leaf length, noSeed: total seed number, PHAH: plant growth in height since aphid introduction. Ellipses: 95% confidence intervals around the centroid for each treatment. Relationship between urbanization and (c) PC1, representing increased rosette size and lower trichome density, and (e) PC2, representing increased allocation of biomass to roots, of the PCA in (a). Relationship between urbanization and (d) PC1, correlated with inflorescence size and plant fitness, and (f) PC2, correlated with rosette size, of the PCA in (b). Lines are based on intercepts and slopes for fixed effects. For model *R* ^2^ *_c_* values and parametric bootstrap *p*‐values of fixed effects see Table 1a (c, e) and Table 1b (d, f). For pairwise differences among slopes and slope significances, see in Table S5 (c, e) and Table S7 (d, f)

**FIGURE 3:**
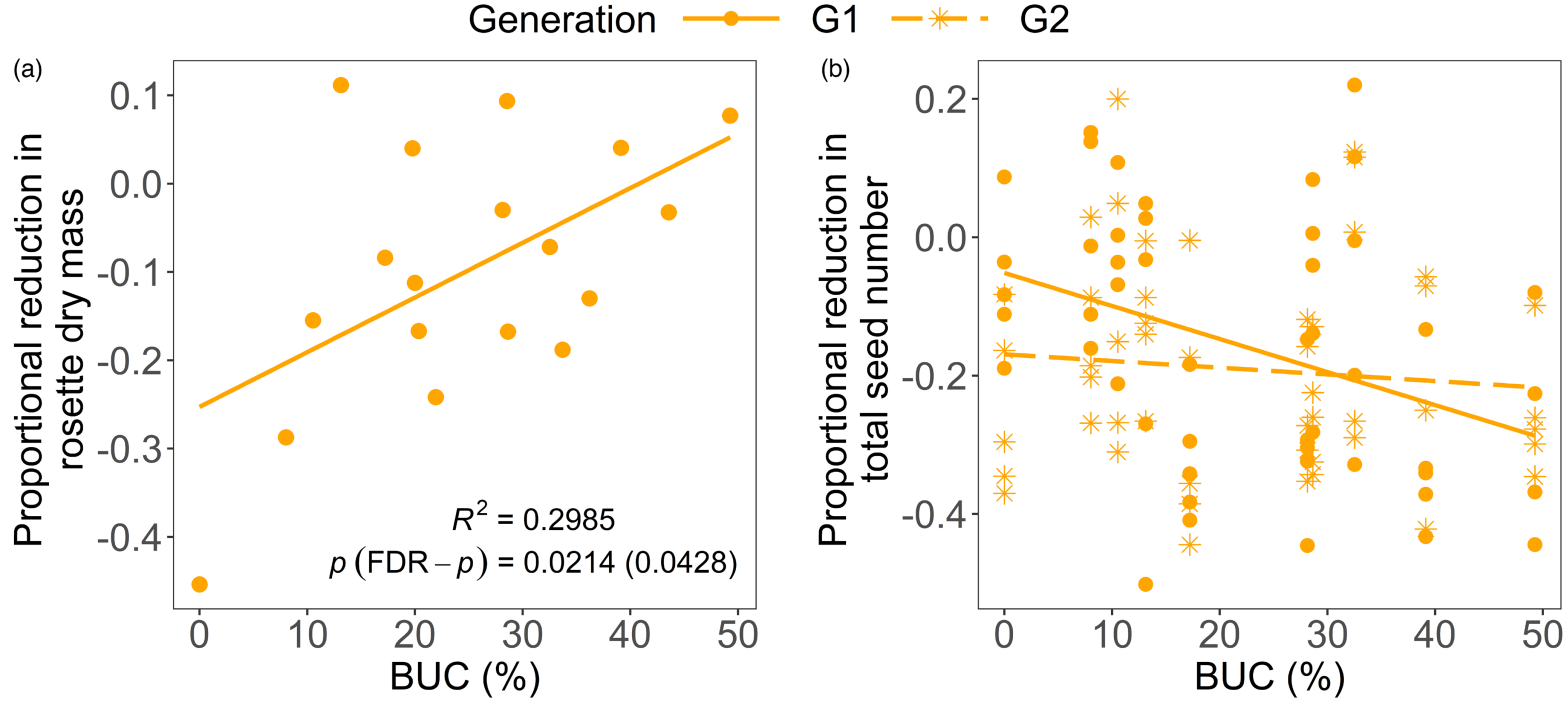
Tolerance of *A*. *thaliana* of (a) rosette leaf dry mass to *P*. *brassicae* and (b) total seed number to *M*. *persicae* in function of (a) urbanization (radius: 200 m) and (b) urbanization and plant generation. Tolerance is the proportional reduction (a) between the genotype mean of plants with and without herbivores relative to the latter and (b) within each plant pair between the plant with and without aphids relative to the latter. (b) Model *R* ^2^ *_c_*, estimates and standard errors of fixed effects, parametric bootstrap and FDR‐adjusted *p*‐values for fixed effects, and *post*‐*hoc* slope comparisons and significances for both plant generations are shown in Table S8b

Higher scores along PC1 represented increased whole plant biomass, rosette biomass, leaf area and root biomass and lower trichome density, while those along PC2 corresponded with increased relative allocation of biomass to roots and root biomass (Figure 2a, Table S3). The PC1 hence represents increased plant size and decreased trichome density, and PC2 is mainly related to increased relative allocation of biomass to roots. Plant trait PC1 scores in both control and herbivory groups significantly increased with increasing levels of urbanization of the plants’ mothers’ environment and were weakly suppressed by caterpillar herbivory (Table 1a, Table S5, Figure 2c). Thus, urban plant genotypes grew larger rosettes than rural plants, with larger biomass and leaf area but fewer trichomes and caterpillars somewhat suppressed plant size. Plant trait PC2 scores in neither control nor herbivory treatment varied with urbanization level but were significantly higher in the herbivory treatment (Table 1a, Table S5, Figure 2e). Biomass allocation to roots was similar in urban plant genotypes as in rural plants, yet was higher in caterpillar‐exposed plants (C vs. P, Table S5). There was a near‐significant interaction between the effects of urbanization and caterpillars on PC2 scores (Table 1a), as the lower root biomass allocation of control plants increased to reach a similar value as caterpillar‐exposed plants at high urbanization values (Figure 2e). However, this interaction became non‐significant after False Discovery Rate correction. Growth rate of caterpillars, absolute leaf area consumed and scaled leaf resistance did not vary among rural and urban genotypes (Table S6, Figure S10). Nevertheless, the total dry biomass of urban *A*. *thaliana* lineages was slightly more tolerant to caterpillars than that of plants of more rural descent (Figure S11a), driven by a greater tolerance of the rosette biomass (Figure 3a), as neither root biomass (Figure S11b) nor leaf area tolerance (Figure S11c) was related to urbanization.

**TABLE 1:** Results of linear mixed‐effect models for plant trait components (PC1, PC2) in relation to urbanization (U: 200 m radius), and (a) caterpillar (*P*. *brassicae*: P) herbivory (T) and their interaction, or (b) seed generation (G), aphid (*M*. *persicae*: M) herbivory (T), and their interactions

(a)	PC1		PC2	
*R* ^2^ *_c_*	0.7497		0.3541	
Fixed effects	Estimate ± SE	*p*(FDR‐*p*)	Estimate ± SE	*p*(FDR‐*p*)
Intercept	−1.75 ± 0.81		−0.745 ± 0.355	
U	0.075 ± 0.029	**0.0244 (0.0488)**	0.024 ± 0.013	0.0899 (0.0899)
T (P)	−0.67 ± 0.348	0.0586 (0.0586)	0.811 ± 0.278	**0.0052 (0.0104)**
U × T (P)	0.016 ± 0.012	0.1997 (0.1997)	−0.02 ± 0.01	0.0504 (0.1008)

Random effects	Variance ± SD		Variance ± SD	
L	2.15 ± 1.466		0.315 ± 0.561	
Residual	1.094 ± 1.046		0.701 ± 0.837	

(b)	PC1		PC2	
*R* ^2^ *_c_*	0.8376		0.814	
Fixed effects	Estimate ± SE	*p*(FDR‐*p*)	Estimate ± SE	*p*(FDR‐*p*)
Intercept	1.447 ± 0.8109		−0.5067 ± 0.7367	
U	−0.0395 ± 0.03	0.2599 (0.2599)	0.0436 ± 0.0273	0.1876 (0.2599)
G (G2)	1.3686 ± 0.3558	**0.0028 (0.0056)**	0.4743 ± 0.3729	0.2331 (0.2331)
T (M)	−0.4291 ± 0.5084	0.446 (0.8309)	−0.0991 ± 0.4099	0.8309 (0.8309)
U × G (F2)	−0.0426 ± 0.013	**0.0072 (0.0144)**	−0.0241 ± 0.0137	0.1171 (0.1171)
U × T (M)	−0.0404 ± 0.0189	0.0758 (0.1012)	−0.03 ± 0.0153	0.1012 (0.1012)
G (G2) × T (M)	−1.2824 ± 0.4645	**0.0208 (0.0416)**	−1.0894 ± 0.4333	**0.0449 (0.0449)**
U × G (G2) × T (M)	0.0378 ± 0.0173	0.0601 (0.0717)	0.0361 ± 0.0162	0.0717 (0.0717)

Random effects	Variance ± SD		Variance ± SD	
G × L × Pair	0.1483 ± 0.3851		0.3437 ± 0.5863	
G × L × T	0.0165 ± 0.1282		0.0376 ± 0.1938	
L × T	0.2209 ± 0.47		0.1086 ± 0.3295	
L	1.5272 ± 1.2358		1.2837 ± 1.133	
Residual	0.7079 ± 0.8414		0.4986 ± 0.7061	

Notes

The common garden for (a) used 18 *A*. *thaliana* genotypes (L) grown from seeds collected at locations varying in urbanization level, (b) used 10 of these 18 genotypes of the first (G1) and second (G2) generation. *R* ^2^_*c*_: conditional *R* ^2^, SE: standard error, SD: standard deviation, *p*: parametric bootstrap *p*‐value, FDR‐*p*: adjusted *p*‐value based on false discovery rate. FDR‐corrections were done for each fixed effect across both response variables. Replicates (plants) per T‐L combination: *N* = 5 (sample size =180 (a) and 200 (b)). Pairs of aphid‐exposed and control plants per L per generation: *N* = 5. Significant *p*‐values (*p* < 0.05) are given in bold.

Trichome density was positively related to scaled leaf resistance (Figure S12a). The negative relationship between trichome density and absolute leaf damage (Figure S12b) was in part driven by the fact that the smaller plants, mostly of rural genotypes, which had higher trichome densities (Figure S12c), cannot lose large absolute areas of leaf. Trichome density was not significantly related to caterpillar growth rate (Figure S12d).

### 3.3 Experiment (2): aphid herbivory

Higher PC1 scores indicated heavier stems, more fruits and thus seeds, more branches, heavier shoots, taller plants, but lower rosette leaf biomass, less growth in height since aphid introduction and shorter length of the longest rosette leaf (Figure 2b, Table S3). PC2 scores were positively correlated with length of the longest rosette leaf and of fruits, biomass of shoots, stems, and rosette leaves, plant height, growth in height since aphid introduction, total number of seeds, while negatively with number of branches (Figure 2b, Table S3). PC1 is thus mainly related to increased inflorescence size and fecundity and PC2 mainly represents increase in rosette size.

For both PC1 and PC2 scores, a near‐significant three‐way interaction among the effects of urbanization, plant generation, and aphid treatment was observed (Table 1b), implying that the effects of urbanization on both PCs scores depended on plant generation and aphid treatment. For PC1 scores, only the slopes of control plants from both generations (G1C vs. G2C) were significantly different after False Discovery Rate correction (Table S7a). Except for control plants of the first generation (G1C), thus, PC1 scores of aphid‐treated plants from the first generation (G1M) and both control (G2C) and aphid‐treated (G2M) second‐generation plants significantly decreased with increasing urbanization levels of the (grand)mothers’ environment (Figure 2d, Table S7b). PC1 scores were also higher for control than aphid‐exposed plants along the urbanization gradient (G1C vs. G1M, G2C vs. G2M), except for G1 control plants (G1C) with mothers from more rural areas, whose PC1 scores were more similar to the corresponding aphid‐treated plants (G1M) (Table 1b, Figure 2d). These results show that urban plants and plants exposed to aphids, except for first‐generation control plants, had lighter stems and fewer branches, fruits, and seeds (indicating lower fitness), but grew taller after aphid introduction and had longer and heavier rosette leaves. For PC2 scores, none of the pairwise slope comparisons (Table S7a) or slopes (Table S7b) was statistically significant, suggesting that PC2 scores did not vary with percentage built‐up area in the environment of the mother plants (Table 1b, Figure 2f). PC2 scores tended to be higher in control than aphid‐exposed plants except for first‐generation control plants (G1C) at low urbanization values, where this difference was much smaller (G1C vs. G1M, G2C vs. G2M).

Aphid tolerance of rosette or stem dry mass did not respond to plant generation or urbanization of the ancestral plants’ environment (Table S8a). The urbanization effect on tolerance of the total seed number was significantly negative for the first generation but not significant for the second generation (Figure 3b, Table S8b). The intrinsic growth rate and maximum size of aphid populations did not respond to urbanization or plant generation (Table S9, Figure S13). Plant shoot dry mass had a strongly positive effect on aphid population size (Table S9). Thus, plant tolerance and resistance did not respond to urbanization and plant generation. The exception was a significantly higher tolerance to aphid herbivory in first‐generation rural plants that decreased to a lower tolerance at high urbanization values.

## 4 DISCUSSION

In line with our first hypothesis, aphid densities were higher on urban plants. However, urban *A*. *thaliana* showed no evolution of increased aphid resistance or tolerance, as aphids reduced plant fitness at least as much in urban plants as in rural plants in a common environment. Moreover, urban plants had a higher tolerance to caterpillar herbivory under controlled environmental conditions. Both these observations are inconsistent with our second hypothesis, but this hypothesis is supported by the lower trichome densities of urban plants in the common garden. Below, we discuss our findings in more detail, and speculate on the underlying mechanisms, including possible trade‐offs among different growth and defence traits in the biotic and abiotic urban environment.

### 4.1 Changes in aphid herbivory in the city

Aphid abundance and densities increased with urbanization in the field. This is in line with the previously reported general pattern (Korányi et al., 2021; Parsons & Frank, 2019; Raupp et al., 2009), but some recent work observed no response of aphid abundance and/or density to the amount of impervious cover (Rocha & Fellowes, 2018, 2020). Aphid peak abundances or population growth rates were not related to urbanization of the environment of the plants’ progenitors in a common garden. However, in the first generation, plants from urban genotypes lost relatively more seeds to aphid herbivory than plants from more rural genotypes. This effect disappeared in second‐generation plants, suggesting that plant mothers in more rural environments can increase their seeds’ tolerance against aphids, which are the most common herbivores of *A*. *thaliana* and have been shown to drive its evolution (Züst et al., 2012). In urban plants, however, there seems to be a maternal effect lowering the offspring's aphid tolerance. Moreover, fitness of aphid‐exposed plants tended to be lower than that of control plants. As aphids were much more abundant in the city, the maternal effect in urban plants causing lower aphid tolerance could be a delayed cost of herbivory. However, if aphid herbivory is unpredictable, a reduced tolerance might provide fitness advantages to other stressors, and eventually be adaptive (Burgess & Marshall, 2014).

Urban warming and drought can independently and interactively increase development rate, abundance, and fecundity of herbivorous insects (Dale & Frank, 2014a, 2014b, 2017; Meineke et al., 2013), which may explain why urban aphid populations were larger in the field (Barton & Ives, 2014). Although water stress in plants can enhance aphid performance due to increased nutrient concentrations in the phloem (Cregg & Dix, 2001), *A*. *thaliana* had constant shoot and root moisture along our urbanization gradient (lack of urbanization effect on PC2 scores, Table S4). The increased root growth of urban genotypes may be an adaptation to cope with the urban environment, as root biomass was not higher in urban field sites, indicating higher loss of root biomass in the city. It is possible that resources required for this increased root growth would otherwise be spent on aphid defence, explaining the lower tolerance to aphids of first‐generation urban plants. Other mechanisms beyond the scope of this study, such as the disruption of the abundance and community composition of predators (Korányi et al., 2021) and phenological mismatches between hosts and enemies (Frank & Just, 2020; Meineke et al., 2014), may also explain aphid outbreaks in urban environments.

### 4.2 Evolution of plants in the city

Our common garden experiments indicated that urban *A*. *thaliana* has evolved lower stem biomass and fewer branches, fruits and seeds, but higher rosette biomass. This is in line with studies documenting that urban plants were generally larger in a common garden, but contrasts their findings that they had a higher fitness (Santangelo et al., 2020; Yakub & Tiffin, 2017). Our plants showed a somewhat increased root biomass with increasing urbanization of their mothers’ environment in the absence of caterpillars. Evolution of this trait might have been driven by urbanization, in line with greater root biomass in urban *Crepis sancta* (Lambrecht et al., 2016). However, *A*. *thaliana* exhibited signs of maternal effects, with a much weaker decrease in inflorescence size and seed set in response to urbanization in the first‐generation control plants. This suggests that urban plants may counteract some of the negative effects on their offspring's fitness, for example by providing better resources for the seeds. Herbivory by aphids diminished these maternal effects, possibly because mother plants have to prioritize allocating resources to coping with aphid‐induced stress.

In the field, urban plants had higher aphid densities but did not differ in size from rural plants, while in the common garden plants with urban mothers had higher rosette biomass, regardless of whether aphids were present. This difference in patterns between the natural system and the laboratory could be due to coevolutionary dynamics or to genotype‐by‐environment (G × E) interactions. Increased aphid population densities with increasing urbanization may imply evolution of herbivores in response to urbanization, which warrants further investigation. Another explanation is that the evolved potential for larger size was not expressed in urban sites due to other growth‐limiting factors in cities such as a limited amount or quality of soil.

### 4.3 Urban plant adaptation to herbivore pressure

Leaf trichomes can efficiently defend against chewing herbivores (Handley et al., 2005; Sletvold et al., 2010). We found a potential genetic basis (Mauricio, 1998) for lower trichome density of urban plants, which was positively related to scaled leaf resistance. Trichome density was not related to larval performance in our experiment. Handley et al. (2005) explained that natural selection for phytophagous insects may favour traits that facilitate egg survival rather than larvae performance. Trichomes can enable *A*. *thaliana* to resist infestation by aphids, but at a growth cost (Sato et al., 2019; Züst et al., 2011). Later‐stage plants often do not show this resistance effect, because aphids favour flowering stems and buds, which have few and no trichomes, respectively (Sato et al., 2019). In addition, in our experiment aphids lowered the fitness of second‐generation plants and caused a proportional decrease in total seed number in paired control and aphid‐exposed plants, indicating real fitness costs of aphid herbivory.

Together, these results indicate that the potential evolution of low trichome density in urban genotypes was not related to defence against aphids or specialist caterpillars. The high cost of trichome production (Mauricio, 1998; Sletvold et al., 2010) may have resulted in its abandonment to free up resources needed to deal with more stressful growing conditions in the city. Trade‐offs with other defences such as secondary chemical compounds may also explain such patterns (Brachi et al., 2015), but this is unlikely as glucosinolate concentrations were not correlated with urbanization levels in the field (Table S10). Alternatively, a lower abundance of other, possibly generalist, leaf‐chewing herbivores in the city may have lowered the need for costly trichomes. Although we observed very few herbivores other than aphids across the urban‐rural gradient, other species including leaf chewers may have been present earlier in the season. *A*. *thaliana* overwinters as rosette and is thus available when *P*. *brassicae* caterpillars start appearing in March. However, caterpillar abundance only peaks in July, when most *A*. *thaliana* plants have set seed and died. Thus, plants and caterpillars co‐occur in the field when caterpillar densities are still relatively low. Furthermore, *P*. *brassicae* may favour other Brassicaceae with more biomass to feed on than the small *A*. *thaliana*. Plant tolerance may be more profitable for inhibiting herbivore damage than resistance (Núñez‐farfán et al., 2007). Indeed, urban plants showed a higher tolerance to caterpillars of the rosette biomass, which may have compensated for a lower trichome density. Whether this tolerance shift is due to trait evolution in response to *P*. *brassicae*, to other chewing herbivores, or to maternal effects, remains to be investigated.

However, first‐generation offspring of urban plants displayed a somewhat lower tolerance of the total seed number to *M*. *persicae*, hinting at a maternal effect with an immediate fitness cost. Like the lower trichome density, the lower aphid tolerance may trade off with the increased caterpillar tolerance. This seems unlikely given that aphids are much more numerous herbivores of this plant species. It seems more likely that maternal resources otherwise invested in aphid tolerance are more needed to cope with other, likely abiotic stressors in urban environments.

### 4.4 Outlook

As our dependence on urban ecosystems will inevitably rise, not only the ecological but also the evolutionary consequences of urbanization need to be understood. Furthermore, urban environments act as sentinels of future climate change effects and may represent hubs for evolutionary processes that may preadapt both hosts and consumers to future conditions outside our cities (Lahr et al., 2018). This may help safeguard ecosystem services, if urban preadaptations increase future plant performance, but also counter them, if pests equally preadapt.

## CONFLICT OF INTEREST

The authors declare no conflict of interest.

## Supporting information

Supplementary Material

## Data Availability

Raw data for this study are available at the Dryad Digital Repository: https://doi:10.5061/dryad.f1vhhmgzf.
