# Supplementary material for: Phenotypic and genotypic divergence of plant–herbivore interactions along an urbanization gradient
Source: Evol Appl. 2022 Apr 25;15(5):865–77. doi: 10.1111/eva.13376 (PMC9108311; doi:10.1111/eva.13376)
Supplement: Supplementary file 1 — Supplementary Material [file EVA-15-865-s001.docx]

**Phenotypic and genotypic divergence of plant-herbivore interactions**

**along an urbanization gradient**

Jiao Qu^1*, 2^, Dries Bonte^2^, Martijn L. Vandegehuchte^2, 3^

**Supplementary material**

**Appendix S1. Details of plant traits measured in the field survey**

For each plant, the number of conspecific plants within 1 m^2^ centered on this plant was recorded as a measure of local plant density. Plant height was measured from the soil surface to the top of the plant to the nearest mm with a tape measure. To investigate invertebrates inhabiting plant shoots, plant aboveground vegetative tissue was clipped at ground level and immediately stored in a labelled plastic bag. This method may have induced some bias against organisms that can quickly move away from plants upon disturbance. However, we were able to work quickly in bagging these small plants. We thus assume such bias to have been minimal. To collect plant roots, we excavated up to approximately 20 g (fresh weight) of rhizosphere soil containing roots and placed it in a plastic bag, which was preserved in a cooler box for transport. Both aboveground and belowground samples were transported to the laboratory and stored in a fridge at 4 °C for a maximum of 3 days before further processing.

In the lab, aboveground invertebrates were separated from shoots and preserved in 70% ethanol for identification. Fresh shoot weight was weighed to the nearest 0.001 g. Preliminary analyses revealed that out of 104 samples, 39 did not harbor any invertebrates. As nearly all invertebrates found in the remaining samples were aphids, for a large part *Myzus persicae* (Sulzer) and *Macrosiphum euphorbiae* (Thomas), they were distinguished from other invertebrates and counted and treated as a single group for analysis in this study. Afterwards, the number of matured fruits was counted and the length of six randomly selected fruits was measured with a digital caliper to the nearest 0.01 mm. We used total seed production as the appropriate measure of fitness for a selfing species such as *A. thaliana* (Heil & Baldwin, 2002). This total seed production was estimated by the product of the number of fruits and the mean fruit length of six fruits. Fruit (i.e., silique) length has been shown to be a reliable proxy for the number of seeds in one fruit (Roux, Gasquez, & Reboud, 2004).

Roots were gently separated from the soil, washed to remove any soil attached to them, blotted gently with a soft paper towel to remove any free surface moisture, and freshly weighed to the nearest 0.001 g. Roots and shoots were oven-dried at 50 °C and soil at 105 °C for at least 72 h and weighed to the nearest 0.001 g. Total plant dry biomass was the sum of shoot and root dry biomass and then the ratio of root to total biomass was calculated. Finally, water contents of shoots and roots were calculated as the difference between fresh and dry weight divided by dry weight.

**References**

Heil, M., & Baldwin, I. T. (2002). Fitness costs of induced resistance: emerging experimental support for a slippery concept. *Trends in Plant Science*, *7*, 61–67.

Roux, F., Gasquez, J., & Reboud, X. (2004). The dominance of the herbicide resistance cost in several *Arabidopsis thaliana* mutant lines. *Genetics*, *166*, 449–460.

**Appendix S2. Details of plant growing and insect herbivore rearing conditions**

In all experiments, seeds were surface-sterilized in a 3% sodium hypochlorite solution and then exposed to stratification at 4°C in the dark for 3 days. Three to nine seeds (depending on availability) of each genotype were sown in each of 10 plastic pots (6 cm top diameter × 4 cm bottom diameter × 5.5 cm height) that were filled with sterilized standard potting soil and already water saturated. Pots were then randomly organized in trays and kept under common conditions (16-hr light, ca. 22 ± 2 °C and 45 ± 10 % relative humidity). Pots in trays were bottom-watered three times per week. Positions of trays and individual pots in trays were randomized every 3 days to minimize any positional effects. After 7 days of establishment, seedlings in each pot were thinned to one of the best individuals.

Eggs of *Pieris brassicae* L. (large cabbage white; Lepidoptera: Pieridae) and *Myzus persicae* (green peach aphid; Hemiptera: Aphididae) were supplied by the Laboratory of Entomology, Wageningen University & Research (WUR) and caterpillars and aphids were reared on Brussels sprouts plants (*B. oleracea* var. *gemmifera* cv Cyrus) and radish plants (*Raphanus sativus* L.), respectively, in cages in the greenhouse under the same conditions as described above.

**Appendix S3. Testing for autocorrelation of model residuals: rationale and methodology**

Variables measured on plants along the rural-urban gradient in the field could potentially display spatial autocorrelation, with samples closer to one another in space having more similar values of plant traits or aphid abundance. This could be due to explanatory variables that themselves exhibit spatial autocorrelation, such as the level of urbanization, but also other, unmeasured variables. Linear models assume independence of errors. In our case this means that after taking into account the variation in response variables explained by the local level of urbanization, the errors should not be spatially autocorrelated for this assumption to be met. We investigated this potential issue for all our linear models predicting plant trait PC1 and PC2 as well as aphid abundance from levels of urbanization measured within each of our eight buffer radii around field sampling locations. We used spline correlograms (‘ncf’ package), which estimate spatial dependence of model Pearson residuals as continuous function of spatial distance among samples based on their latitude and longitude coordinates. If the 95% pointwise bootstrap confidence interval of the spline correlogram includes zero, spatial autocorrelation of the tested variable is not statistically significant. Before constructing the spline correlograms, latitude and longitude coordinates were converted into Universal Transverse Mercator (UTM) through the system of wgs84 by the function ‘spTransform’ (‘rgdal’ package).

**Table S1.** Percentage built-up cover calculated in radii with eight different diameters around 18 maternal *Arabidopsis thaliana* plants used in the common garden experiment. All 18 *A. thaliana* genotypes (L) of the first generation were exposed to *Pieris brassicae*, while 10 genotypes of the first and second generation were exposed to *Myzus persicae*, marked in bold.

| Genotype  (L) | Built-up cover (%) at 8 radii around the sampled plant | | | | | | | |
| --- | --- | --- | --- | --- | --- | --- | --- | --- |
|  | 50 m | 100 m | 200 m | 400 m | 800 m | 1600 m | 2400 m | 3200 m |
| **L109** | 0 | 0 | 0 | 1.1856 | 2.0894 | 6.4752 | 11.1538 | 12.1329 |
| **L52** | 4.2748 | 16.6913 | 10.5306 | 5.1524 | 2.6867 | 7.5061 | 12.5305 | 15.5504 |
| **L82** | 5.8401 | 8.1367 | 13.1207 | 16.9651 | 14.9088 | 16.2559 | 13.8912 | 14.5228 |
| **L40** | 6.0963 | 12.1878 | 8.0290 | 10.7131 | 16.0480 | 15.9760 | 15.9074 | 16.6451 |
| **L84** | 10.4740 | 13.1030 | 17.2206 | 15.9969 | 11.5395 | 12.9857 | 13.7137 | 14.0822 |
| L83 | 19.0217 | 18.5366 | 20.3574 | 16.3315 | 12.1931 | 17.0158 | 19.5567 | 18.2695 |
| L99 | 19.7957 | 20.2311 | 20.0049 | 24.6000 | 25.6682 | 23.7628 | 21.8286 | 20.8489 |
| L94 | 20.5265 | 30.0768 | 33.7087 | 33.5718 | 26.1396 | 19.1972 | 16.5178 | 16.2627 |
| L31 | 22.5993 | 21.3239 | 19.7729 | 25.5311 | 25.9999 | 21.4306 | 19.0169 | 18.5082 |
| **L59** | 26.3037 | 25.2522 | 28.6375 | 38.3678 | 34.9228 | 37.8555 | 31.4337 | 26.0227 |
| L25 | 26.9008 | 23.6111 | 28.5588 | 24.7493 | 32.4758 | 25.7698 | 23.5796 | 22.1453 |
| L95 | 35.3691 | 25.4891 | 21.9355 | 21.4287 | 22.3059 | 16.5422 | 13.9695 | 14.3186 |
| **L56** | 35.6355 | 37.4447 | 28.1437 | 26.3210 | 30.2893 | 28.4574 | 26.3672 | 23.1352 |
| L64 | 35.6640 | 39.3483 | 43.5782 | 46.9171 | 45.0099 | 37.1609 | 32.1654 | 26.6523 |
| **L68** | 41.1814 | 42.1632 | 49.2717 | 55.3457 | 49.9266 | 38.4193 | 33.1524 | 26.3140 |
| L8 | 42.4114 | 39.1295 | 36.2082 | 31.7827 | 33.7676 | 31.5464 | 29.8142 | 24.7134 |
| **L71** | 45.0472 | 46.5750 | 39.1330 | 34.8191 | 39.2031 | 29.7835 | 25.9807 | 22.4317 |
| **L1** | 46.0540 | 40.3977 | 32.5233 | 33.8780 | 32.3721 | 28.0964 | 27.6393 | 23.6358 |

**Table S2.** An exponential growth curve was fitted for each aphid (*M. persicae*) population (Figure S4) feeding on ten *A. thaliana* genotypes (L) of the first (G1) and second (G2) plant generation.

| Genotype | Generation | Replicate | r | *R*^2^ | Generation | Replicate | r | *R*^2^ |
| --- | --- | --- | --- | --- | --- | --- | --- | --- |
| L1 | G1 | R1 | 0.2167 | 0.8333 | G2 | R1 | 0.2472 | 0.9714 |
|  |  | R11 | 0.2316 | 0.7816 |  | R11 | 0.2188 | 0.6480 |
|  |  | R12 | 0.2159 | 0.9203 |  | R5 | 0.2658 | 0.9260 |
|  |  | R3 | 0.2310 | 0.7953 |  | R6 | 0.2624 | 0.9698 |
|  |  | R7 | 0.1953 | 0.7293 |  | R8 | 0.2046 | 0.7341 |
| L109 | G1 | R1 | 0.2075 | 0.9590 | G2 | R10 | 0.2099 | 0.8977 |
|  |  | R11 | 0.1847 | 0.9487 |  | R11 | 0.1636 | 0.9756 |
|  |  | R13 | 0.1749 | 0.9691 |  | R13 | 0.2057 | 0.9604 |
|  |  | R2 | 0.2093 | 0.9562 |  | R4 | 0.2122 | 0.8741 |
|  |  | R8 | 0.2131 | 0.9803 |  | R5 | 0.1962 | 0.9592 |
| L40 | G1 | R11 | 0.1926 | 0.7814 | G2 | R1 | 0.1971 | 0.8625 |
|  |  | R12 | 0.2264 | 0.9050 |  | R10 | 0.2307 | 0.9290 |
|  |  | R5 | 0.2248 | 0.8178 |  | R12 | 0.2074 | 0.9029 |
|  |  | R6 | 0.2170 | 0.8456 |  | R3 | 0.1923 | 0.7470 |
|  |  | R8 | 0.2488 | 0.9181 |  | R9 | 0.2307 | 0.9443 |
| L52 | G1 | R1 | 0.2485 | 0.9695 | G2 | R12 | 0.1697 | 0.8205 |
|  |  | R10 | 0.2293 | 0.7270 |  | R13 | 0.2106 | 0.9216 |
|  |  | R11 | 0.2621 | 0.9790 |  | R2 | 0.2170 | 0.9348 |
|  |  | R3 | 0.2729 | 0.7386 |  | R7 | 0.2080 | 0.8497 |
|  |  | R6 | 0.2424 | 0.7147 |  | R9 | 0.2237 | 0.9382 |
| L56 | G1 | R11 | 0.2265 | 0.8830 | G2 | R1 | 0.1643 | 0.9822 |
|  |  | R13 | 0.2661 | 0.9919 |  | R13 | 0.2436 | 0.9563 |
|  |  | R3 | 0.1869 | 0.8748 |  | R3 | 0.2406 | 0.7731 |
|  |  | R4 | 0.2457 | 0.9814 |  | R6 | 0.2199 | 0.8881 |
|  |  | R7 | 0.2349 | 0.7740 |  | R9 | 0.2537 | 0.9282 |
| L59 | G1 | R1 | 0.1920 | 0.8382 | G2 | R10 | 0.1521 | 0.7906 |
|  |  | R11 | 0.1662 | 0.9632 |  | R2 | 0.2073 | 0.8742 |
|  |  | R13 | 0.1598 | 0.8462 |  | R4 | 0.2087 | 0.7335 |
|  |  | R4 | 0.1915 | 0.7690 |  | R5 | 0.2016 | 0.7805 |
|  |  | R6 | 0.2189 | 0.8718 |  | R9 | 0.1800 | 0.9099 |
| L68 | G1 | R1 | 0.2143 | 0.8341 | G2 | R10 | 0.2299 | 0.8389 |
|  |  | R12 | 0.2167 | 0.7650 |  | R2 | 0.2201 | 0.7462 |
|  |  | R2 | 0.2305 | 0.8137 |  | R3 | 0.2617 | 0.7981 |
|  |  | R4 | 0.2071 | 0.7783 |  | R5 | 0.2392 | 0.7907 |
|  |  |  |  |  |  | R9 | 0.2162 | 0.8122 |
| L71 | G1 | R12 | 0.2434 | 0.9216 | G2 | R1 | 0.2227 | 0.7471 |
|  |  | R13 | 0.2452 | 0.9070 |  | R12 | 0.1881 | 0.6563 |
|  |  | R3 | 0.2485 | 0.7847 |  | R13 | 0.2103 | 0.8885 |
|  |  | R5 | 0.2654 | 0.7659 |  | R8 | 0.2470 | 0.8991 |
|  |  | R9 | 0.2305 | 0.7501 |  |  |  |  |
| L82 | G1 | R1 | 0.1992 | 0.7631 | G2 | R11 | 0.1969 | 0.9353 |
|  |  | R12 | 0.1876 | 0.8626 |  | R12 | 0.2016 | 0.8710 |
|  |  | R13 | 0.2194 | 0.9077 |  | R13 | 0.1932 | 0.8589 |
|  |  | R3 | 0.2153 | 0.8665 |  | R2 | 0.2198 | 0.8454 |
|  |  | R9 | 0.1985 | 0.7449 |  | R7 | 0.2149 | 0.9055 |
| L84 | G1 | R1 | 0.2373 | 0.7340 | G2 | R1 | 0.2177 | 0.5804 |
|  |  | R12 | 0.2295 | 0.7263 |  | R11 | 0.2412 | 0.8481 |
|  |  | R13 | 0.2629 | 0.8893 |  | R12 | 0.2394 | 0.5732 |
|  |  | R4 | 0.2225 | 0.6447 |  | R5 | 0.2473 | 0.7492 |
|  |  | R6 | 0.1857 | 0.9938 |  | R7 | 0.2300 | 0.7510 |

Notes

The growth constant r acted as a measure of aphid population growth rate. *R*^2^ is the goodness of model fit. Five replicates (R) per genotype per generation were used. One introduced aphid for L68 of G1 and another one for L71 of G2 died and therefore were excluded from the corresponding analyses.

**Table S3.** Pearson correlation coefficients (r) of traits that are significantly correlated (*p* < 0.05) with the two principal components (PC1, PC2) of the PCA on plant traits from field survey and caterpillar and aphid experiment.

| Variable | Pearson’s r | *p* |
| --- | --- | --- |
| **Field survey – PC1** |  |  |
| Shoot biomass | 0.97 | < 0.0001 |
| Whole plant biomass | 0.97 | < 0.0001 |
| Total seed number | 0.96 | < 0.0001 |
| Total fruit number | 0.94 | < 0.0001 |
| Root biomass | 0.89 | < 0.0001 |
| Plant height | 0.74 | < 0.0001 |
| Mean fruit length | 0.44 | < 0.0001 |
| Relative biomass allocation to roots | -0.34 | 0.0004 |
| **Field survey – PC2** |  |  |
| Shoot moisture content | 0.75 | < 0.0001 |
| Mean fruit length | 0.66 | < 0.0001 |
| Root moisture content | 0.44 | < 0.0001 |
| Conspecific plant density | -0.37 | 0.0001 |
| **Experiment (1): caterpillar herbivory – PC1** |  |  |
| Whole plant biomass | 0.98 | < 0.0001 |
| Rosette biomass | 0.97 | < 0.0001 |
| Actual leaf area | 0.96 | < 0.0001 |
| Root biomass | 0.94 | < 0.0001 |
| Leaf trichome density | -0.81 | < 0.0001 |
| **Experiment (1): caterpillar herbivory – PC2** |  |  |
| Relative biomass allocation to roots | 0.997 | < 0.0001 |
| Root biomass | 0.26 | 0.0005 |
| **Experiment (2): aphid herbivory – PC1** |  |  |
| Stem biomass | 0.93 | < 0.0001 |
| Total fruit number | 0.92 | < 0.0001 |
| Total seed number | 0.91 | < 0.0001 |
| Branch number | 0.81 | < 0.0001 |
| Shoot biomass | 0.59 | < 0.0001 |
| Plant height | 0.25 | 0.0004 |
| Rosette biomass | -0.56 | < 0.0001 |
| Growth in height since aphid introduction | -0.50 | < 0.0001 |
| Length of the longest leaf | -0.42 | < 0.0001 |
| **Experiment (2): aphid herbivory – PC2** |  |  |
| Length of the longest leaf | 0.75 | < 0.0001 |
| Shoot biomass | 0.71 | < 0.0001 |
| Rosette biomass | 0.64 | < 0.0001 |
| Plant height | 0.62 | < 0.0001 |
| Fruit length | 0.58 | < 0.0001 |
| Growth in height since aphid introduction | 0.54 | < 0.0001 |
| Total seed number | 0.32 | < 0.0001 |
| Stem biomass | 0.27 | < 0.0001 |
| Branch number | -0.19 | 0.0071 |

**Table S4.** Responses of *A. thaliana* plant trait components (PC1, PC2: simple linear models) and aphid abundance (negative binomial generalized linear model) to urbanization calculated in a radius of 200 meter around sampled plants (sample size = 104).

|  | *R*^2^ | Slope ± SE | Intercept ± SE | *p* (FDR-*p*) | |
| --- | --- | --- | --- | --- | --- |
| PC1 | 0.009 | -0.02 ± 0.0208 | 0.4288 ± 0.5016 | 0.3883 (0.5825) | |
| PC2 | 0.0008 | -0.003 ± 0.0107 | 0.0652 ± 0.2595 | 0.7792 (0.7792) | |
|  | *R*^2^*_c_* | Slope ± SE | Intercept ± SE | χ^2^ test | *p* (FDR-*p*) |
| Aphid abundance | 0.277 | 0.0726 ± 0.0188 | 0.9327 ± 0.5039 | χ^2^_1_ = 11.0015 | **0.0009** (**0.0027**) |
| Covariate: shoot dry mass |  | 1.505 ± 0.8055 |  | χ^2^_1_ = 2.8222 | 0.093 |

Notes

The NB-GLM of aphid abundance included shoot dry mass as a covariate. PC1 is correlated with increases in plant size and fecundity and PC2 with increased moisture and decreased local plant density. *R*^2^*_c_*: conditional *R*^2^, SE: standard error, *p*: parametric bootstrap *p*-value for linear models and type-III Wald Chi^2^ *p*-value for NB-GLM, FDR-*p*: adjusted *p*-value based on false discovery rate. FDR-corrections were done across all response variables. Significant *p*-values (*p* < .05) are given in bold.

**Table S5.** Results of *post-hoc* test for pairwise comparisons between slopes and slope differences from zero, of the relationship between urbanization and two plant trait components (PC1, PC2) for control and caterpillar (*Pieris brassicae*) herbivory treatment (P: *P. brassicae* caterpillars, C: control) in a common garden experiment.

|  | PC1 | | | |  | PC2 | | | |
| --- | --- | --- | --- | --- | --- | --- | --- | --- | --- |
|  | Estimate **±** SE | *df* | *t* test | *p* |  | Estimate **±** SE | *df* | *t* test | *p* |
| *Contrast* |  |  |  |  |  |  |  |  |  |
| C – P | -0.0159 **±** 0.0125 | 164 | -1.276 | 0.2039 |  | 0.02 **±** 0.01 | 164 | 2.001 | **0.0471** |
| *Slope* |  |  |  |  |  |  |  |  |  |
| C | 0.0753 **±** 0.0306 | 22.2 | 2.461 | **0.0221** |  | 0.0236 **±** 0.0133 | 28.2 | 1.769 | 0.0878 |
| P | 0.0912 **±** 0.0306 | 22.2 | 2.983 | **0.0068** |  | 0.0036 **±** 0.0133 | 28.2 | 0.267 | 0.7913 |

Notes

Eighteen *A. thaliana* genotypes of the first generation were exposed to caterpillar herbivory. PC1 is related increased plant size and decreased trichome density, and PC2 to increased relative allocation of biomass to roots. SE: standard error, *df*: degree of freedom. Significant *p*-values (*p* < .05) are given in bold.

**Table S6.** Output of linear (mixed-effect) models that test plant resistance traits against caterpillars (*Pieris brassicae*: P) in relation to urbanization (U: 200 m radius) in a common garden experiment using eighteen *Arabidopsis thaliana* genotypes (L) grown from seeds whose mothers grew at locations varying in urbanization level. Caterpillar growth rate was fitted by linear models.

|  | Absolute leaf damage | |  | Scaled leaf resistance | |  | Caterpillar growth rate | |
| --- | --- | --- | --- | --- | --- | --- | --- | --- |
| *R*^2^/*R*^2^*_c_* | 0.4368 | |  | 0.4368 | |  | 0.0003 | |
| Fixed effects | Estimate ± SE | *p*  (FDR-*p*) |  | Estimate ± SE | *p*  (FDR-*p*) |  | Estimate ± SE | *p*  (FDR-*p*) |
| Intercept | 143.603 ± 38.988 |  |  | 0.719 ± 0.076 |  |  | 0.669 ± 0.025 |  |
| U | 1.885 ± 1.392 | 0.2211 (0.3347) |  | -0.004 ± 0.003 | 0.2231 (0.3347) |  | 1.5e-4 ± 9e-4 | 0.8685 (0.8685) |
| Random effects | Variance ± SD |  |  | Variance ± SD |  |  |  |  |
| L | 4251 ± 65.2 |  |  | 0.016 ± 0.127 |  |  |  |  |
| Residual | 6212 ± 78.82 |  |  | 0.024 ± 0.154 |  |  |  |  |

Notes

*R*^2^*_c_*: conditional *R*^2^, SE: standard error, SD: standard deviation, *p*: parametric bootstrap *p*-value, FDR-*p*: adjusted *p*-value based on false discovery rate. The *p*-values were FDR-adjusted separately for each of the fixed effects across all response variables. Replicates (plants) per treatment: *N* = 5 (sample size = 90).

**Table S7.** Results of *post-hoc* test for (a) pairwise comparisons among slopes and (b) slope differences from zero, of the relationship between urbanization and two plant trait components (PC1, PC2) for different combinations of aphid (*M. persicae*) herbivory treatment (M: *M. persicae* aphids, C: control) and plant generation in a common garden experiment.

| (a) | PC1 | | | |  | PC2 | | | |
| --- | --- | --- | --- | --- | --- | --- | --- | --- | --- |
| Contrast | Estimate **±** SE | *df* | *t* test | *p*  (FDR-*p*) |  | Estimate **±** SE | *df* | *t* test | *p*  (FDR-*p*) |
| G1C – G1M | 0.0404 **±** 0.0211 | 19.2 | 1.909 | 0.0713  (0.1426) |  | 0.03 **±** 0.0171 | 22 | 1.749 | 0.0942  (0.3567) |
| G1C – G2C | 0.0426 **±** 0.0145 | 24.6 | 2.94 | **0.007**  (**0.042**) |  | 0.0241 **±** 0.015 | 28.8 | 1.607 | 0.1189  (0.3567) |
| G1C – G2M | 0.0452 **±** 0.0217 | 21.6 | 2.083 | **0.0493**  (0.1426) |  | 0.018 **±** 0.0188 | 32.2 | 0.958 | 0.345  (0.6645) |
| G1M – G2C | 0.0023 **±** 0.0219 | 22.2 | 0.104 | 0.9183  (0.9183) |  | -0.0059 **±** 0.0189 | 33 | -0.31 | 0.7588  (0.7588) |
| G1M – G2M | 0.0048 **±** 0.015 | 27.2 | 0.322 | 0.7496  (0.9183) |  | -0.012 **±** 0.0154 | 31.3 | -0.777 | 0.443  (0.6645) |
| G2C – G2M | 0.0026 **±** 0.0211 | 19.1 | 0.122 | 0.9043  (0.9183) |  | -0.0061 **±** 0.0171 | 21.7 | -0.359 | 0.7232  (0.7588) |

| (b) | PC1 | | | |  | PC2 | | | |
| --- | --- | --- | --- | --- | --- | --- | --- | --- | --- |
| Slope | Estimate **±** SE | *df* | *t* test | *p*  (FDR-*p*) |  | Estimate **±** SE | *df* | *t* test | *p*  (FDR-*p*) |
| G1C | -0.0395 **±** 0.0336 | 16.1 | -1.176 | 0.2567  (0.2567) |  | 0.0435 **±** 0.0305 | 15.9 | 1.43 | 0.1721  (0.6632) |
| G1M | -0.0798 **±** 0.0337 | 16.4 | -2.367 | **0.0305**  **(0.0407)** |  | 0.0136 **±** 0.0306 | 16.2 | 0.444 | 0.6632  (0.6632) |
| G2C | -0.0821 **±** 0.0336 | 16.2 | -2.443 | **0.0264**  **(0.0407)** |  | 0.0194 **±** 0.0305 | 16 | 0.637 | 0.533  (0.6632) |
| G2M | -0.0847 **±** 0.0337 | 16.3 | -2.515 | **0.0228**  **(0.0407)** |  | 0.0256 **±** 0.0306 | 16.1 | 0.836 | 0.4152  (0.6632) |

Notes

Ten *A. thaliana* genotypes of the first (G1) and second (G2) generation were exposed to aphid herbivory. PC1 was positively correlated with inflorescence size and plant fitness, and PC2 with rosette size. SE: standard error, *df*: degree of freedom, FDR-*p*: adjusted *p*-value based on false discovery rate. FDR-corrections were done for each response variable across all treatments. Significant *p*-values (*p* < .05) are given in bold.

**Table S8.** Results of (a) linear (mixed-effect) models that test tolerance characteristics of *A. thaliana* growth and fitness to herbivory by *M. persicae* in response to urbanization (U), seed generation (G) and their interaction (U × G) with urbanization calculated in a 200 meter radius, and (b) *post-hoc* pairwise comparisons between slopes and slope differences from zero for the two generations.

| (a) | *R*^2^/*R*^2^*_c_* | Term group | Term | Estimate ± SE | *p* (FDR-*p*) |
| --- | --- | --- | --- | --- | --- |
| Proportional reduction in rosette dry mass | 0.0315 | Fixed effects | Intercept | 0.2017 **±** 0.1083 |  |
|  |  |  | U | -0.0054 **±** 0.0041 | 0.0721 (0.0959) |
|  |  |  | G (G2) | -0.2587 **±** 0.1525 | 0.0937 (0.1406) |
|  |  |  | U × G (G2) | 0.0089 **±** 0.0058 | 0.1261 (0.1892) |
| Proportional reduction in stem dry mass | 0.3403 | Fixed effects | Intercept | -0.0262 **±** 0.0581 |  |
|  |  |  | U | -0.0043 **±** 0.0022 | 0.0959 (0.0959) |
|  |  |  | G (G2) | -0.095 **±** 0.0562 | 0.1479 (0.1479) |
|  |  |  | U × G (G2) | 0.0025 **±** 0.0021 | 0.2911 (0.2911) |
|  |  | Random effects |  | Variance ± SD |  |
|  |  |  | G × L | 0.0011 **±** 0.033 |  |
|  |  |  | L | 0.0053 **±** 0.0725 |  |
|  |  |  | Residual | 0.0175 **±** 0.1323 |  |
| Proportional reduction in total number of seeds | 0.2452 | Fixed effects | Intercept | -0.0515 **±** 0.0542 |  |
|  |  |  | U | -0.0048 **±** 0.002 | **0.049** (0.0959) |
|  |  |  | G (G2) | -0.1177 **±** 0.0544 | **0.0402** (0.1206) |
|  |  |  | U × G (G2) | 0.0038 **±** 0.0021 | 0.073 (0.1892) |
|  |  | Random effects |  | Variance ± SD |  |
|  |  |  | L | 0.0042 **±** 0.0652 |  |
|  |  |  | Residual | 0.0215 **±** 0.1465 |  |

| (b) | Proportional reduction in rosette dry mass | | | |  | Proportional reduction in stem dry mass | | | |  | Proportional reduction in total number of seeds | | | |
| --- | --- | --- | --- | --- | --- | --- | --- | --- | --- | --- | --- | --- | --- | --- |
|  | Estimate **±** SE | *df* | *t* test | *p* |  | Estimate **±** SE | *df* | *t* test | *p* |  | Estimate **±** SE | *df* | *t* test | *p* |
| *Contrast* |  |  |  |  |  |  |  |  |  |  |  |  |  |  |
| G1 – G2 | -0.0089 **±** 0.0058 | 94 | -1.546 | 0.1253 |  | -0.0025 **±** 0.0024 | 12.7 | -1.059 | 0.3092 |  | -0.0038 **±** 0.0021 | 90.4 | -1.83 | 0.0705 |
| *Slope* |  |  |  |  |  |  |  |  |  |  |  |  |  |  |
| G1 | -0.0054 **±** 0.0041 | 94 | -1.319 | 0.1904 |  | -0.0043 **±** 0.0024 | 20 | -1.782 | 0.0899 |  | -0.0048 **±** 0.0022 | 23 | -2.154 | **0.042** |
| G2 | 0.0035 **±** 0.004 | 94 | 0.863 | 0.3902 |  | -0.0018 **±** 0.0024 | 19.5 | -0.761 | 0.4558 |  | -0.001 **±** 0.0022 | 22 | -0.44 | 0.6639 |

Notes

Ten *A. thaliana* genotypes (L) of the first (G1) and second (G2) generation were exposed to *M. persicae* in a common garden experiment. Tolerance is the proportional reduction within each pair of plants between the plant with aphids and that without aphids relative to the latter. Tolerance in rosette dry mass was fitted by linear models, while in stem dry mass and total number of seeds by linear mixed-effect models. *R*^2^*_c_*: conditional *R*^2^, SE: standard error, SD: standard deviation, *p* (a): parametric bootstrap *p*-value, FDR-*p*: adjusted *p*-value based on false discovery rate, *df*: degree of freedom. FDR-*p* values for all variables were adjusted separately for each of the fixed effects across all response variables. Replicates (plants) per treatment: *N* = 5. Significant *p*-values (*p* < .05) are given in bold.

**Table S9.** Results of a linear mixed-effect model of aphid (*M. persicae*) population growth rate and of a negative binomial generalized mixed model of aphid population peak that relate to seed generation (G), urbanization (U: 200 m radius) and their interaction (U × G), as determined in a common garden experiment employing ten *A. thaliana* genotypes (L) of the first (G1) and second (G2) generation.

|  | Aphid population growth rate | |  | Aphid population peak | | |
| --- | --- | --- | --- | --- | --- | --- |
| *R*^2^*_c_* | 0.3493 | |  | 0.7196 | | |
| Fixed effects | Estimate ± SE | *p* (FDR-*p*) |  | Estimate ± SE | χ^2^ test | *p* (FDR-*p*) |
| Intercept | 0.2157 ± 0.0089 |  |  | 6.4036 ± 0.097 |  |  |
| U | 0.0002 ± 0.0004 | 0.6294 (0.6294) |  | 0.1458 ± 0.0965 | χ^2^_1_ = 2.2858 | 0.1306 (0.2612) |
| G (G2) | -0.0126 ± 0.0107 | 0.3195 (0.3705) |  | -0.0458 ± 0.0511 | χ^2^_1_ = 0.8019 | 0.3705 (0.3705) |
| U × G (G2) | 0.0004 ± 0.0004 | 0.4212 (0.4576) |  | 0.0377 ± 0.0508 | χ^2^_1_ = 0.5516 | 0.4576 (0.4576) |
| Shoot dry mass |  |  |  | 0.129 ± 0.0201 | χ^2^_1_ = 41.0495 | **< 0.0001** |
| Random effects | Variance ± SD |  |  | Variance ± SD |  |  |
| G × L | 8e-5 ± 0.0089 |  |  | 0.0068 ± 0.0828 |  |  |
| L | 1e-4 ± 0.0106 |  |  | 0.081 ± 0.2847 |  |  |
| Residual | 4e-4 ± 0.021 |  |  |  |  |  |

Notes

Dry weight of shoots was included as a covariate in the model of aphid population peak. *R*^2^*_c_*: conditional *R*^2^, SE: standard error, SD: standard deviation, *p*: parametric bootstrap *p*-value for linear mixed-effect model and type-III chi-square for negative binomial generalized mixed model, FDR-*p*: adjusted *p*-value based on false discovery rate. FDR-*p*-values for all variables were corrected separately for each of the fixed effects across both response variables. Replicates (plants) per treatment: *N* = 5. Significant *p*-values (*p* < .05) are given in bold.

**Table S10.** Outputs of generalized linear models testing for the relationships between individual glucosinolate (GLS) compounds of *A. thaliana* and urbanization levels in the field survey.

| Variables | Family | Scale (m) | *R*^2^*_c_* | Estimates | Residual deviance | *df* | Dispersion parameter | *p* |
| --- | --- | --- | --- | --- | --- | --- | --- | --- |
| PRO | Binomial | 3200 | 0.0677 | -0.1286 | 101.47 | 98 |  | **0.0115** |
|  | Gamma | 100 | 0.3416 | 0.0613 | 25.426 | 21 | 1.6154 | 0.0829 |
| GBC | Binomial | 50 | 0.032 | 0.0207 | 135.75 | 98 |  | 0.09 |
|  | Gamma | 100 | 0.0182 | -0.0098 | 69.12 | 48 | 1.2957 | 0.5356 |
| HIR | Binomial | 2400 | 0.009 | 0.0307 | 109.34 | 98 |  | 0.3488 |
|  | Gamma | 800 | 0.2113 | -0.0333 | 35.864 | 22 | 3.5856 | 0.2357 |
| GLS1 | Binomial | 3200 | 0.1064 | -0.1274 | 123.56 | 98 |  | **0.0014** |
|  | Gamma | 200 | 0.2641 | 0.0546 | 80.617 | 37 | 2.3737 | 0.0718 |
| GLS2 | Binomial | 1600 | 0.0304 | 0.036 | 120.49 | 98 |  | 0.068 |
|  | Gamma | 50 | 0.2366 | -0.0481 | 61.809 | 29 | 3.1023 | 0.0873 |
| GLS4 | Binomial | 3200 | 0.0158 | -0.057 | 113.07 | 98 |  | 0.214 |
|  | Gamma | 50 | 0.156 | 0.0159 | 6.4105 | 24 | 0.2233 | **0.0362** |
| Total GLS | Binomial | 3200 | 0.0231 | -0.0437 | 100.59 | 98 |  | 0.1379 |
|  | Gamma | 3200 | 0.0233 | -0.0403 | 214.01 | 77 | 3.6252 | 0.3428 |

Notes

Their presences are modelled with a binomial distribution and their concentrations with a gamma distribution. Listed individual compounds are those present in at least 20 samples (field-collected sample size = 104, GLS-tested sample size = 100. Four samples were lost due to improper storage.). Results of the best-fitting model at the scale with largest conditional *R*^2^ value (*R*^2^*_c_*) across eight spatial scales (50–3200 m radii) are shown. PRO: Progoitrin GLS, GBC: Glucobrassicin GLS, HIR: Glucohirsutin GLS. GLS1/GLS2/GLS4: Unidentified GLS, *df*: degree of freedom. Significant *p*-values (*p* < .05) are given in bold.

**Figure S1.** Study area of Ghent in northwest Belgium. All symbols indicate 104 *Arabidopsis thaliana* samples in the field survey. Eighteen genotypes of the first generation (G1) were exposed to *Pieris brassicae*, and ten genotypes of the first and second (G2) generation to *Myzus persicae* in the common garden experiments. The lower-right inset shows the eight nested spatial scales (50–3200 m radii) in which the percentage of built-up cover was determined.


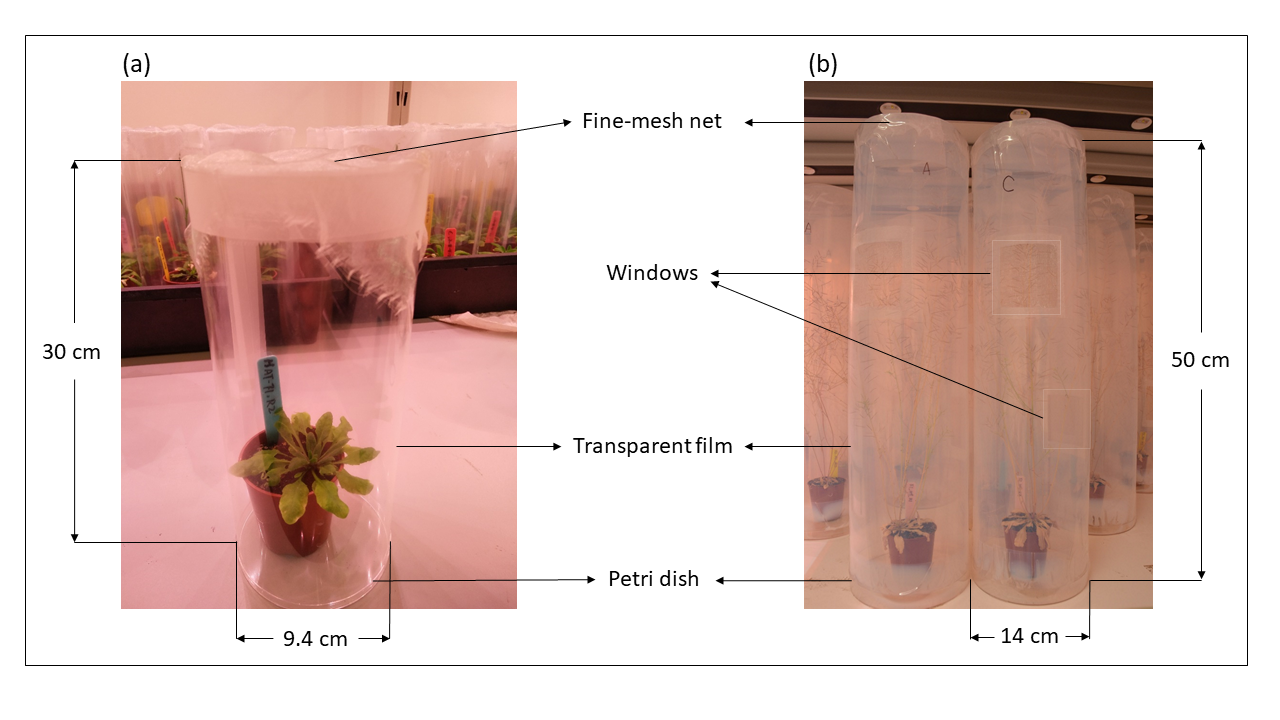


**Figure S2.** Photograph of experimental cages. Cages were employed to keep (a) caterpillars and (b) aphids constrained to the individual plants assigned to the herbivore treatment. Plants in control treatments also were placed in identical cages to ensure they were exposed to the same cage effect. Each aphid cage had two square windows (side length: 6 cm) at 1/3 and 2/3 heights of the cage and on opposite sides to facilitate airflow through the cage.


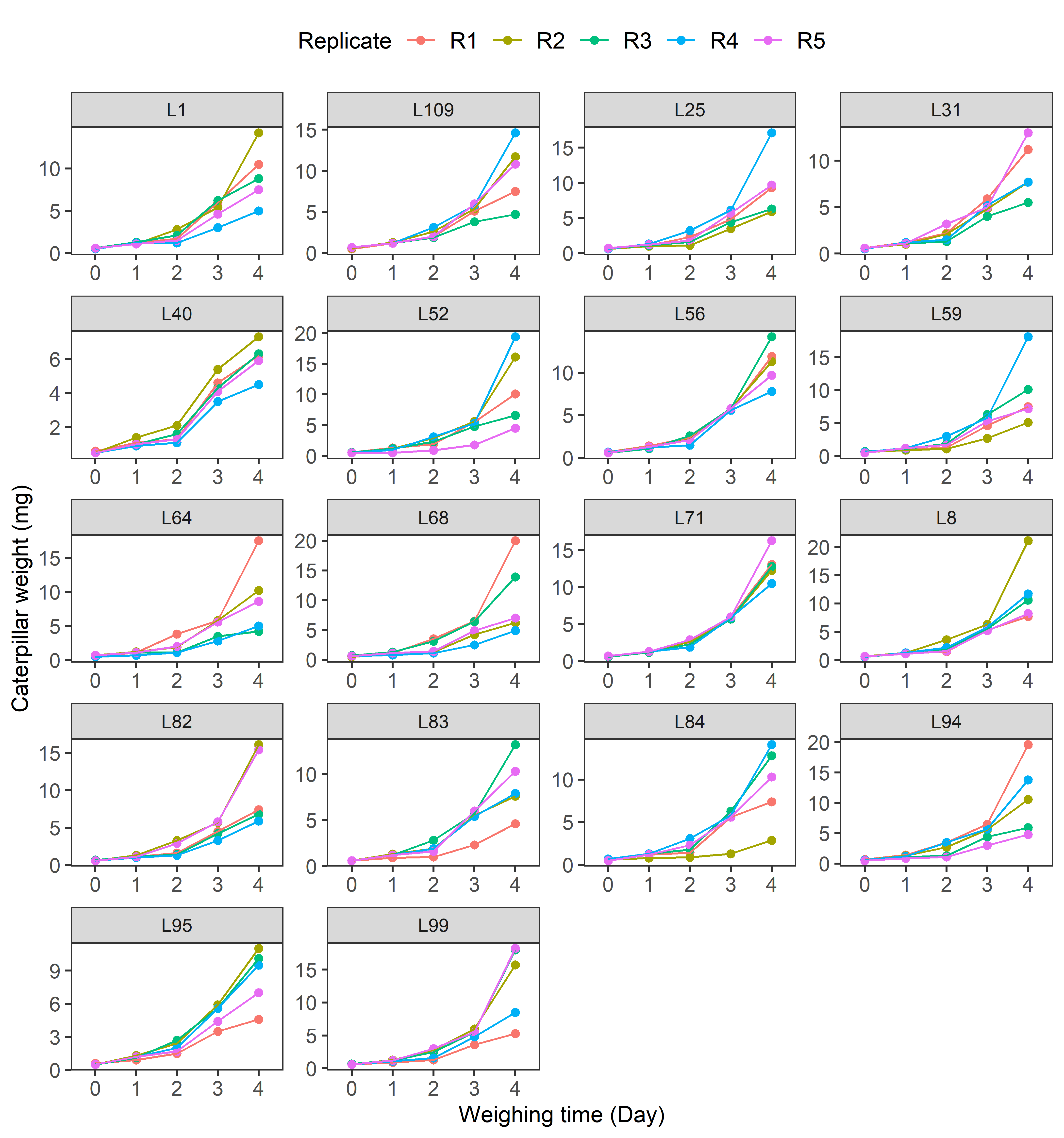


**Figure S3.** Weight change of caterpillars (*Pieris brassicae*) feeding on each genotype (L) of *Arabidopsis thaliana* during 5 continuous days. Weights of the caterpillar on each individual replicate (R) plant are connected with straight lines. Replicates (plants) per treatment: *N* = 5.


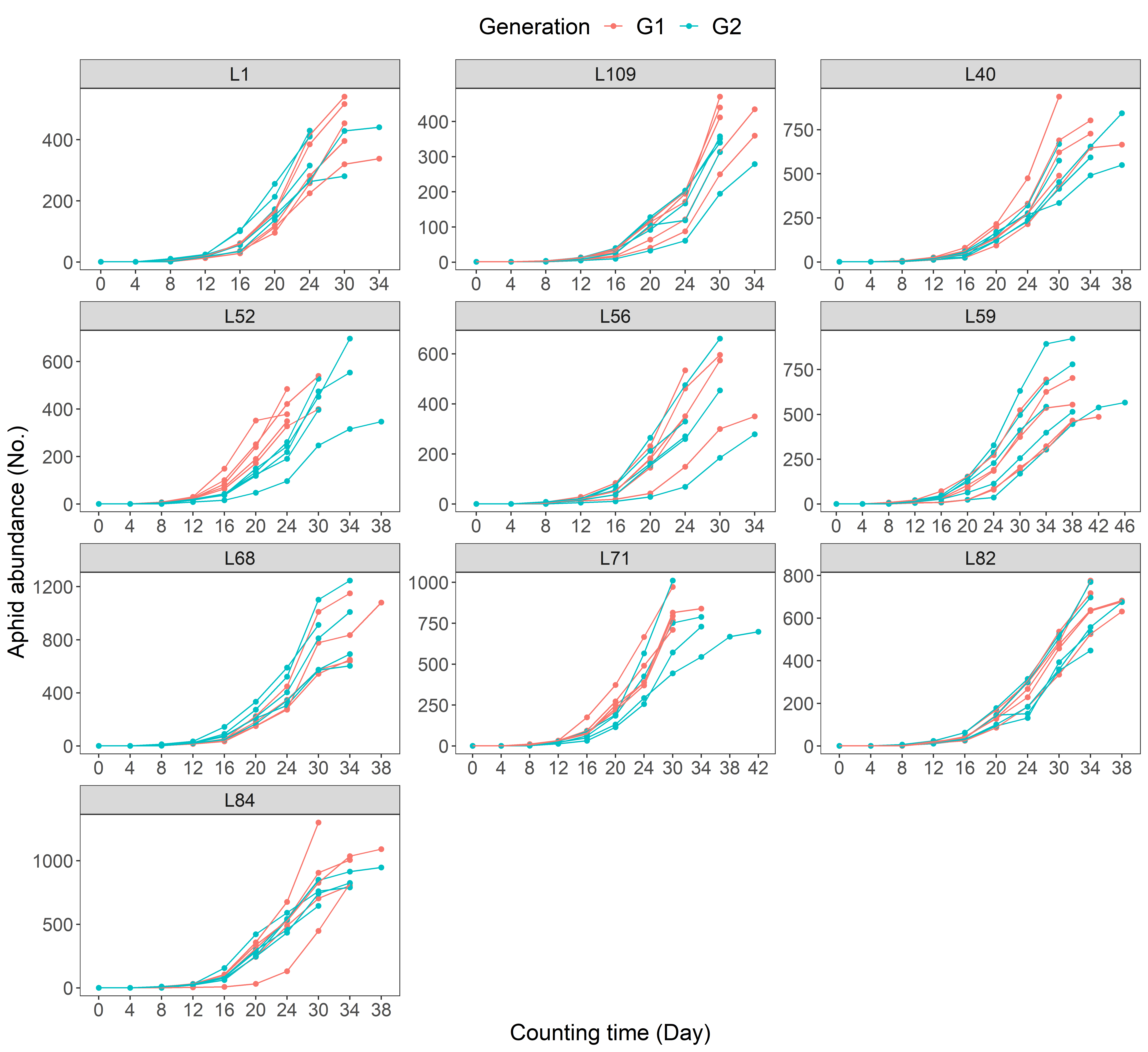


**Figure S4.** Aphid (*Myzus persicae*) population growth on each genotype (L) of *Arabidopsis thaliana* for the first (G1) and second (G2) generation until the population peak. Numbers of aphids counted on each individual replicate plant are connected with straight lines. Replicates (plants) per treatment: *N* = 5.


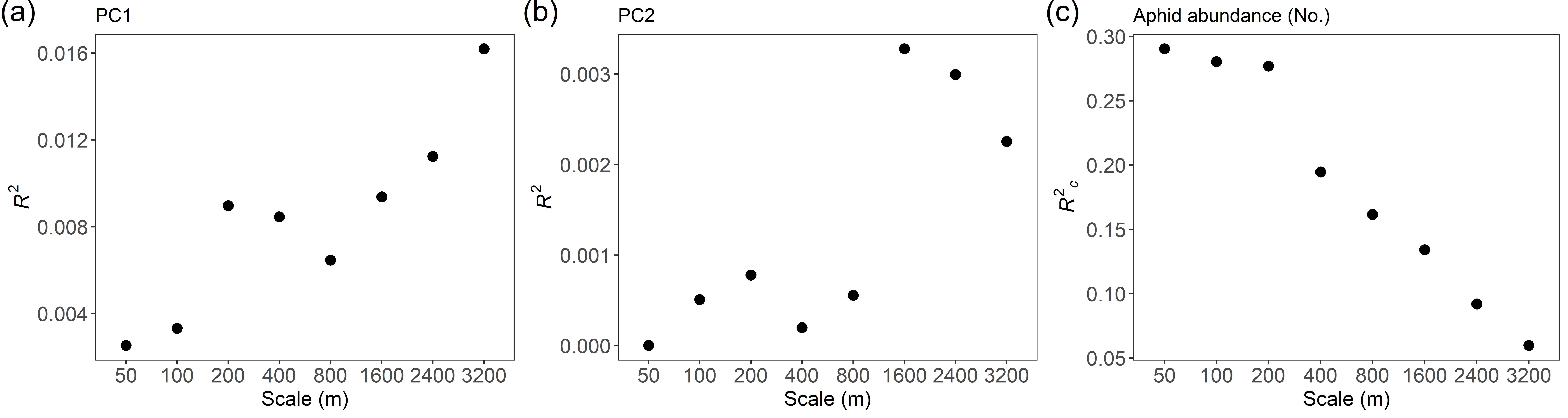


**Figure S5.** Visualization of model (conditional) R-square (*R*^2^/*R*^2^*_c_*) as a function of the radius around the sampled plants in which urbanization was calculated for models testing the effect of urbanization on (a) PC1, (b) PC2 and (c) aphid abundance in the field survey. PC1 and PC2 were fitted with linear models, while aphid abundance with negative binomial generalized linear models including shoot dry biomass as a covariate.





**Figure S6.** Visualization of model (conditional) R-square (*R*^2^/*R*^2^*_c_*) as a function of the radius around the sampled plants in which urbanization was calculated for linear (mixed) models testing urbanization (and herbivory treatment) effects on (a) PC1, (b) PC2, (c) absolute leaf damage, (d) scaled absolute leaf damage, (e) caterpillar growth rate, and (f-i) tolerance in rosette biomass, root biomass, total biomass and total leaf area in the caterpillar lab experiment. Variables in a-e were fitted with linear mixed-effect models, while variables in f-i were fitted with linear models.





**Figure S7.** Visualization of model (conditional) R-square (*R*^2^/*R*^2^*_c_*) as a function of the radius around the sampled plants in which urbanization was calculated for (generalized) linear mixed models testing urbanization, plant generation (and herbivory treatment) effects on (a) PC1, (b) PC2, (c) aphid population growth rate, (d) aphid population peak, (e-g) tolerance of dry rosette mass, dry stem mass and total number of seeds in the aphid lab experiment. Aphid abundance was fitted with generalized linear mixed models including shoot dry mass as a covariate, while other variables were fitted with linear mixed models.


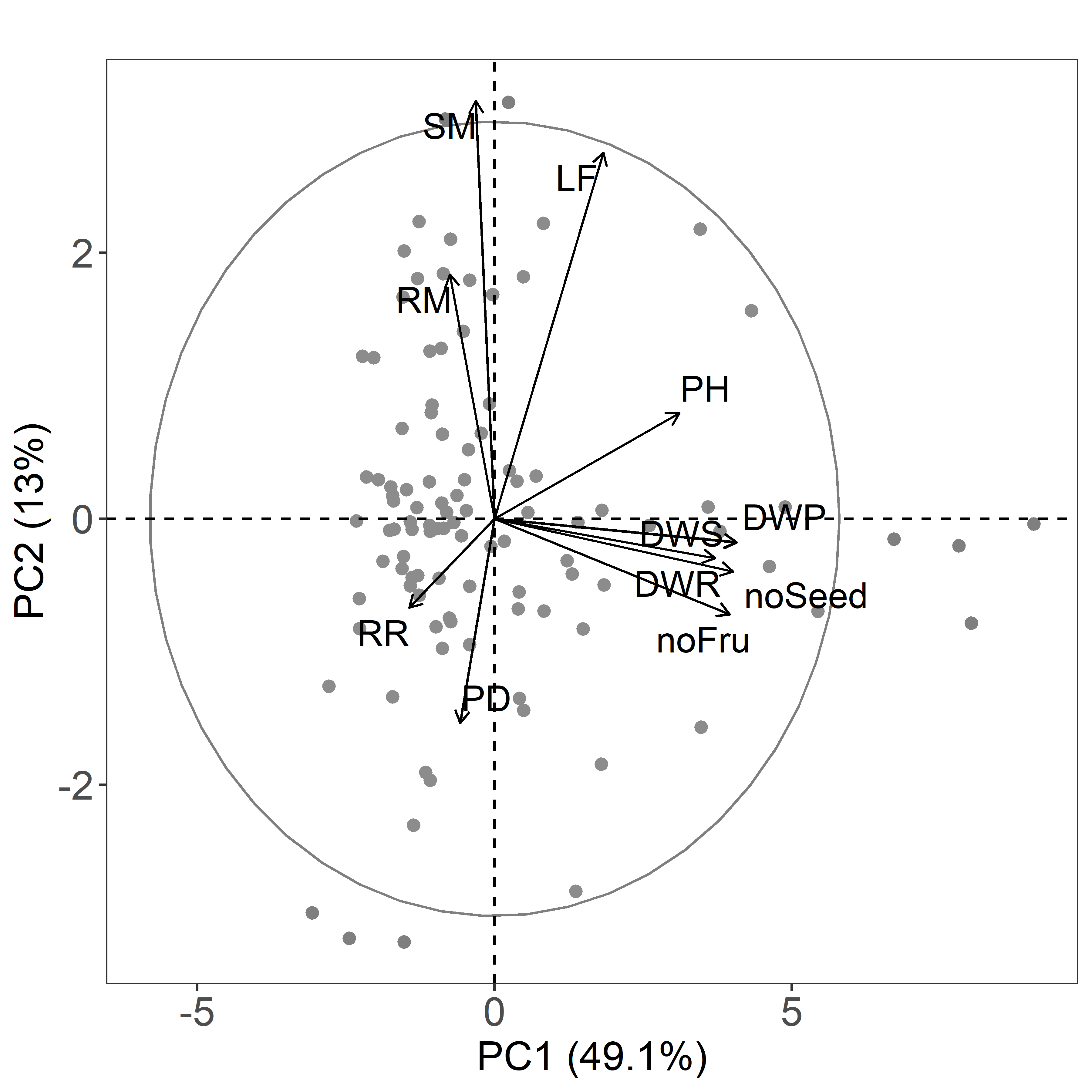


**Figure S8.** PCA summarizing trait variation among *A. thaliana* plants across an urbanization gradient in the field survey. The PCA is based on standardized data on the conspecific plant density within 1 m^2^ square (PD), plant height (PH), dry shoot weight (DWS), dry root weight (DWR), dry total plant weight (DWP), number of fruits (noFru), mean length of fruits (LF), shoot moisture (SM), root moisture (RM), ratio of root to total plant mass (RR), total number of seeds (noSeed). The arrows indicate the coefficients of traits on the two principal components (PC1 and PC2) and hence point in the direction in which values of that trait are maximized. The ellipse indicates 95% confidence intervals around the centroid.

(a) PC1
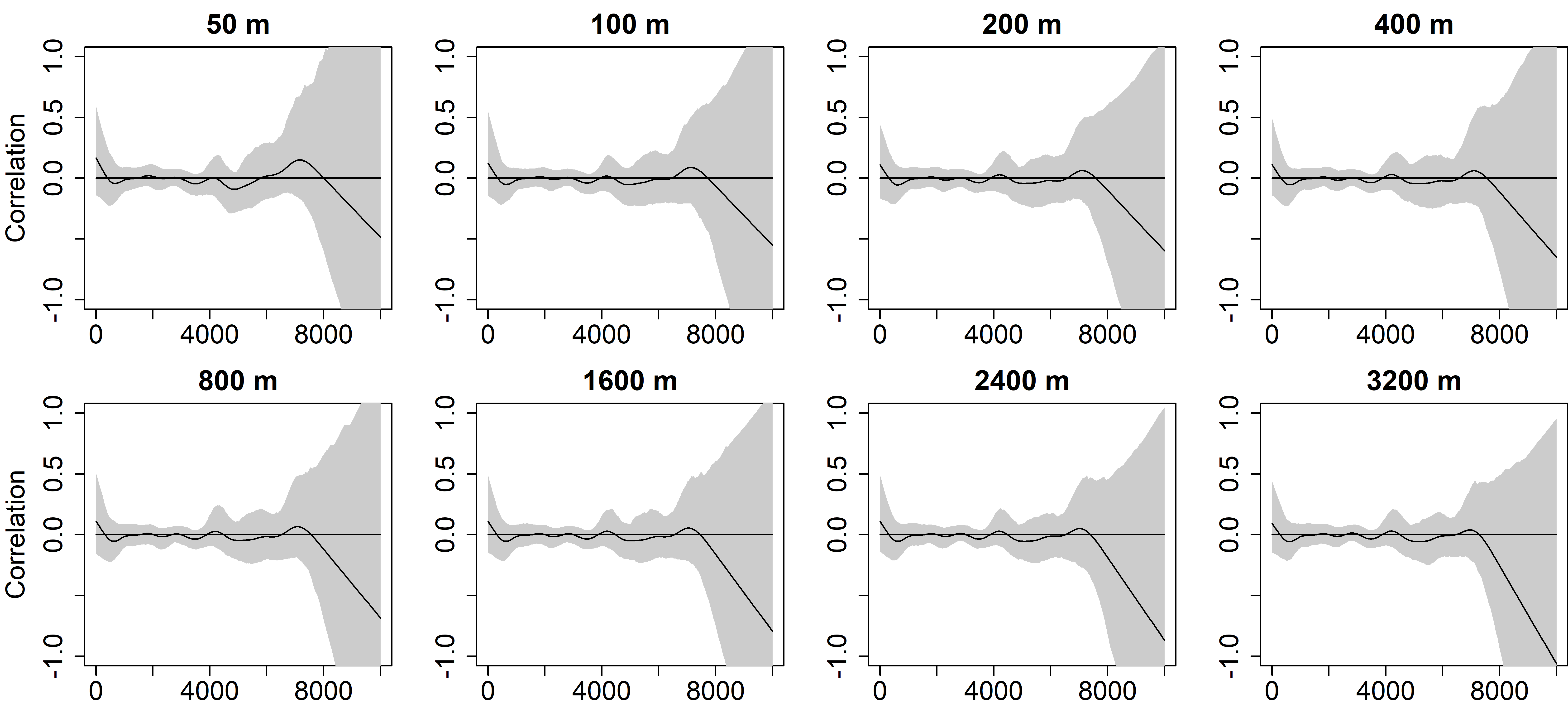


(b) PC2


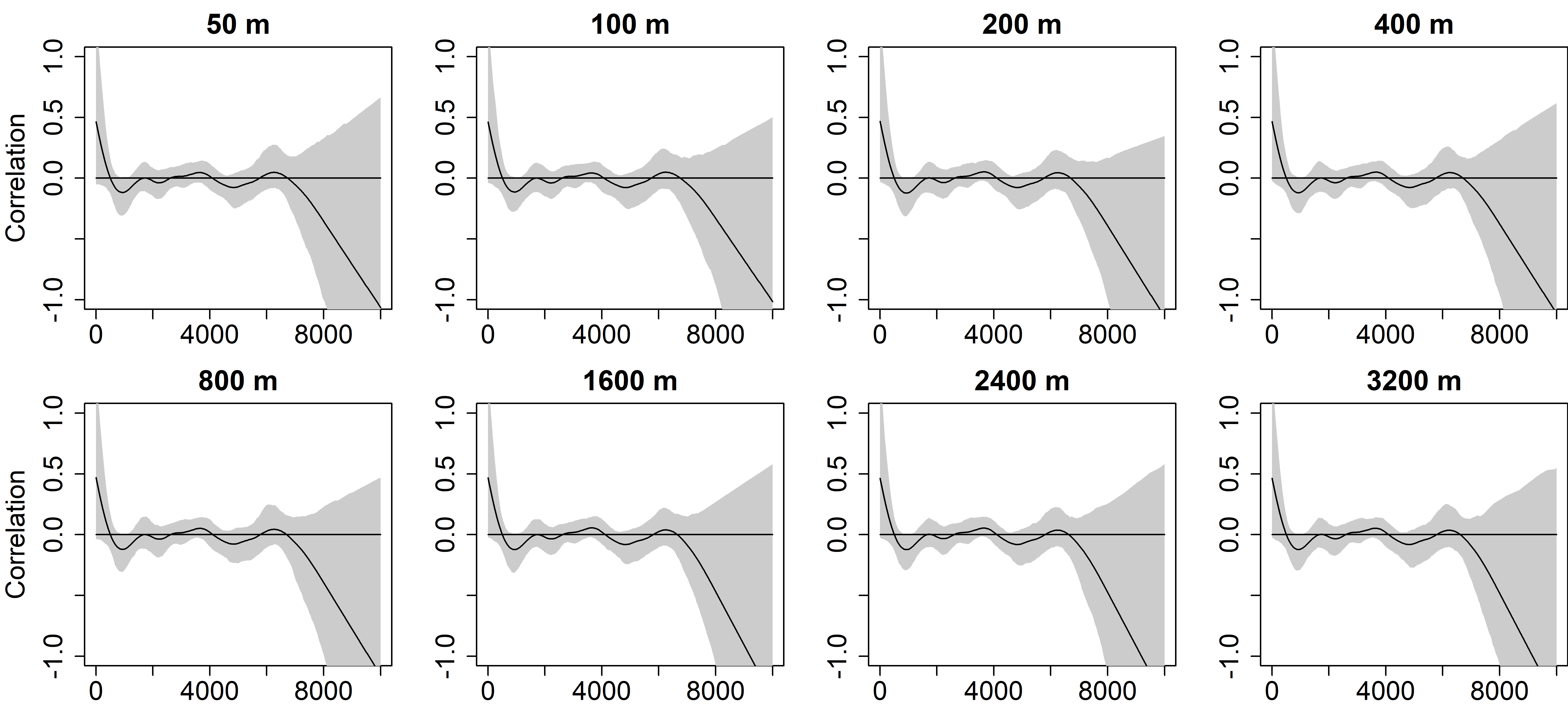


(c) Aphid abundance


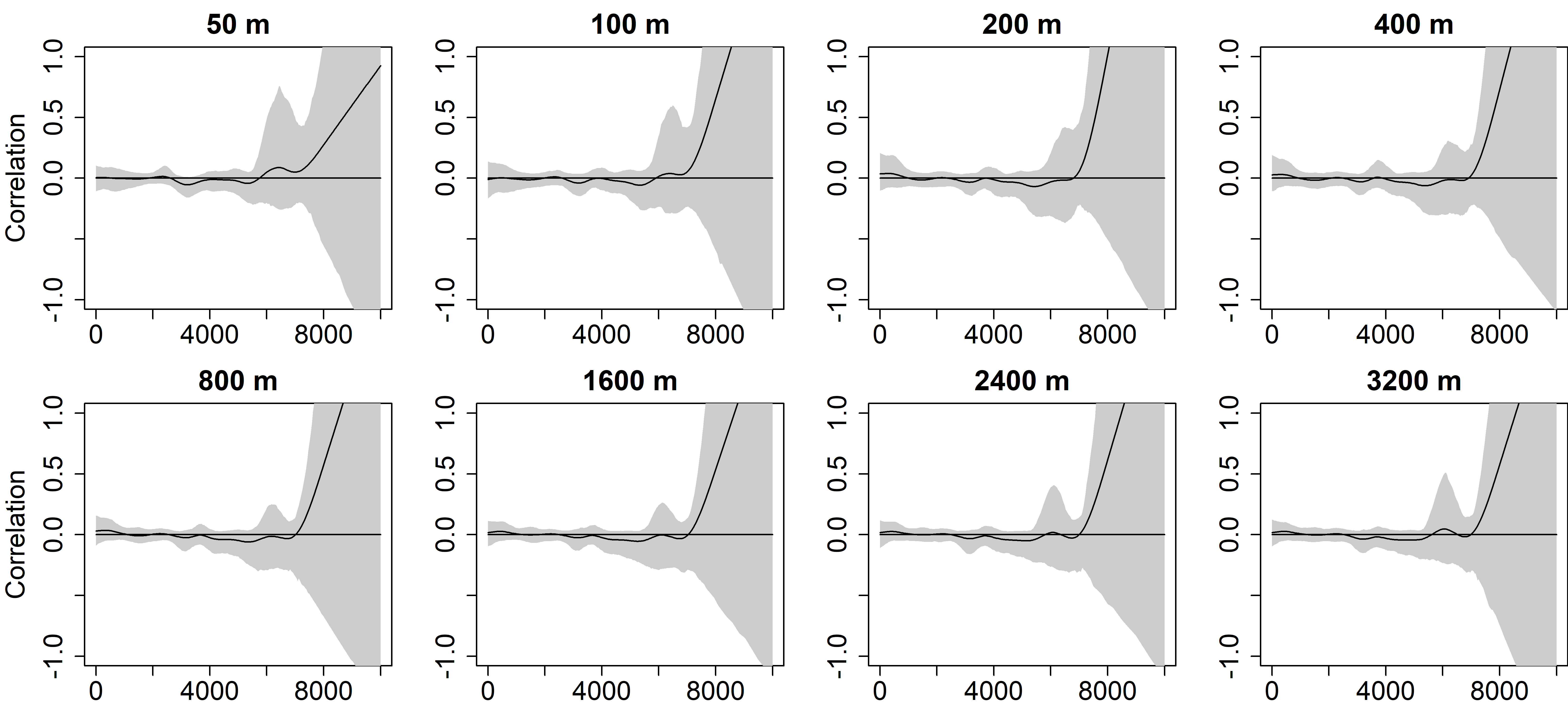


**Figure S9.** Spline correlograms, with 95% pointwise bootstrap confidence intervals, of the Pearson residuals from (generalized) linear models that test for the effects of urbanization values (% BUC) on two key principal components of variation in *Arabidopsis thaliana* traits and aphid abundance at the eight scales (50–3200 m) in the field investigation. The model for aphid abundance included shoot dry mass as a covariate. Each correlogram depicts the degree of spatial autocorrelation of the model residuals in function of varying lag distances.


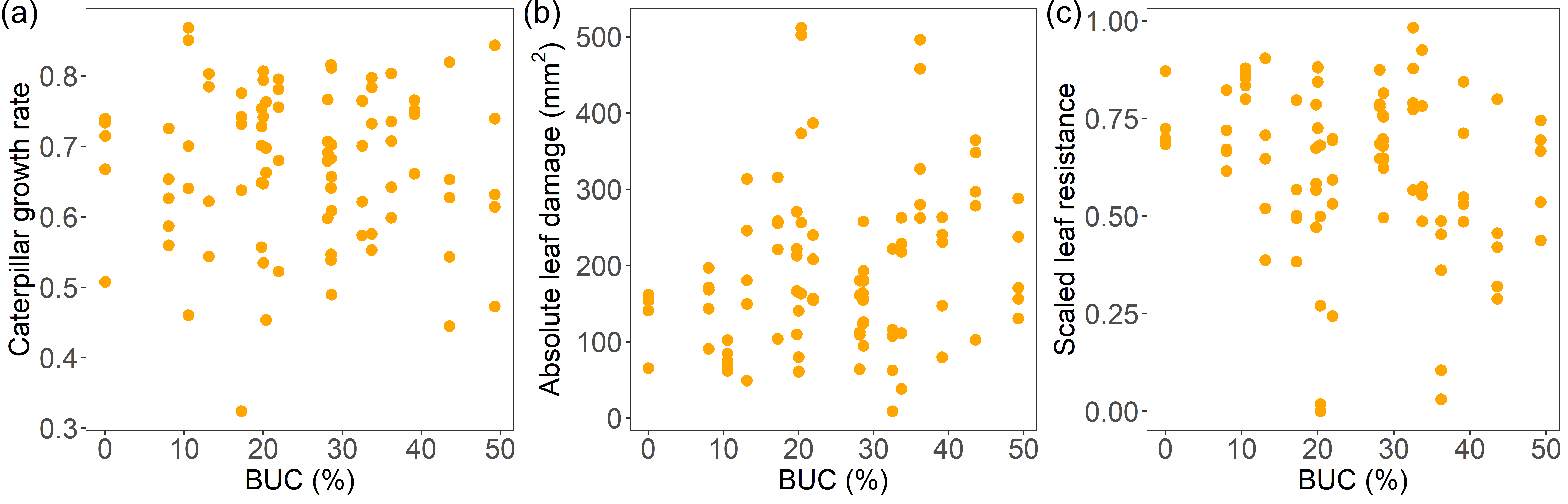


**Figure S10.** Caterpillar (*P. brassicae*) traits as a function of urbanization (200 m radius), as determined in a common garden experiment using eighteen *A. thaliana* genotypes grown from seeds whose mothers grew at locations varying in urbanization level. Raw data of variables were plotted against percentage BUC in a radius of 200 meter around the mother plants. For each variable, model *R*^2^*_c_*, estimates, *p*-values and FDR-*p*-values of the fixed effects are given in Table S5.


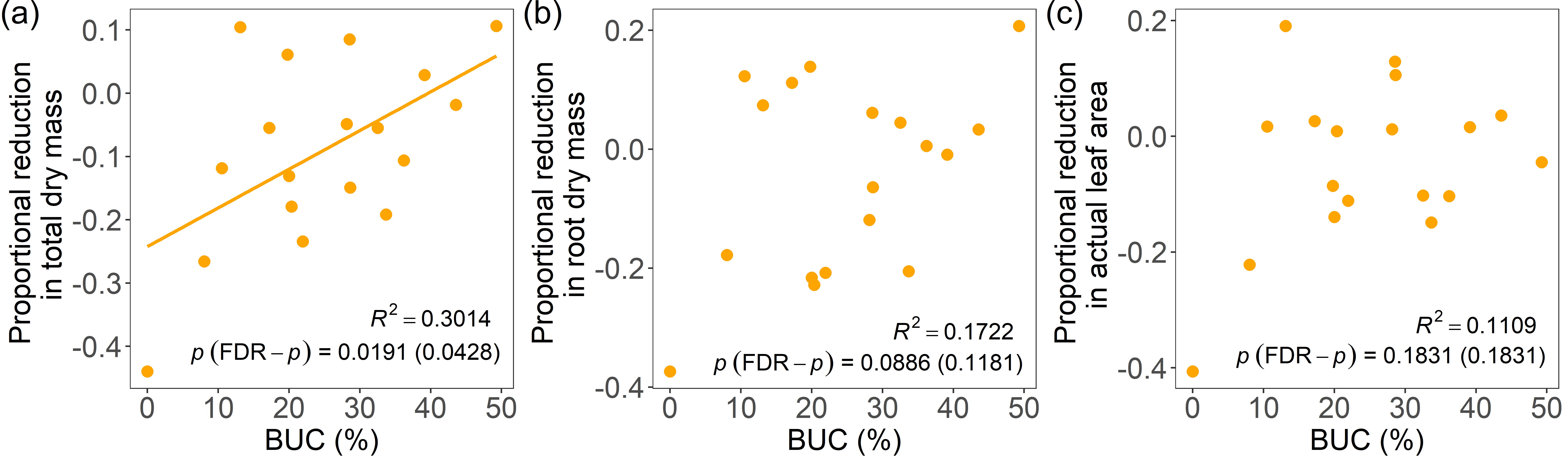


**Figure S11.** Aspects of *A. thaliana* tolerance to herbivory by *P. brassicae* as a linear function of urbanization in a 200 m radius. Model *R*^2^, parametric bootstrap *p*-values and FDR-*p*-values are given, and a straight line was fitted for plot (a) which shows a significant response (*p* < 0.05).


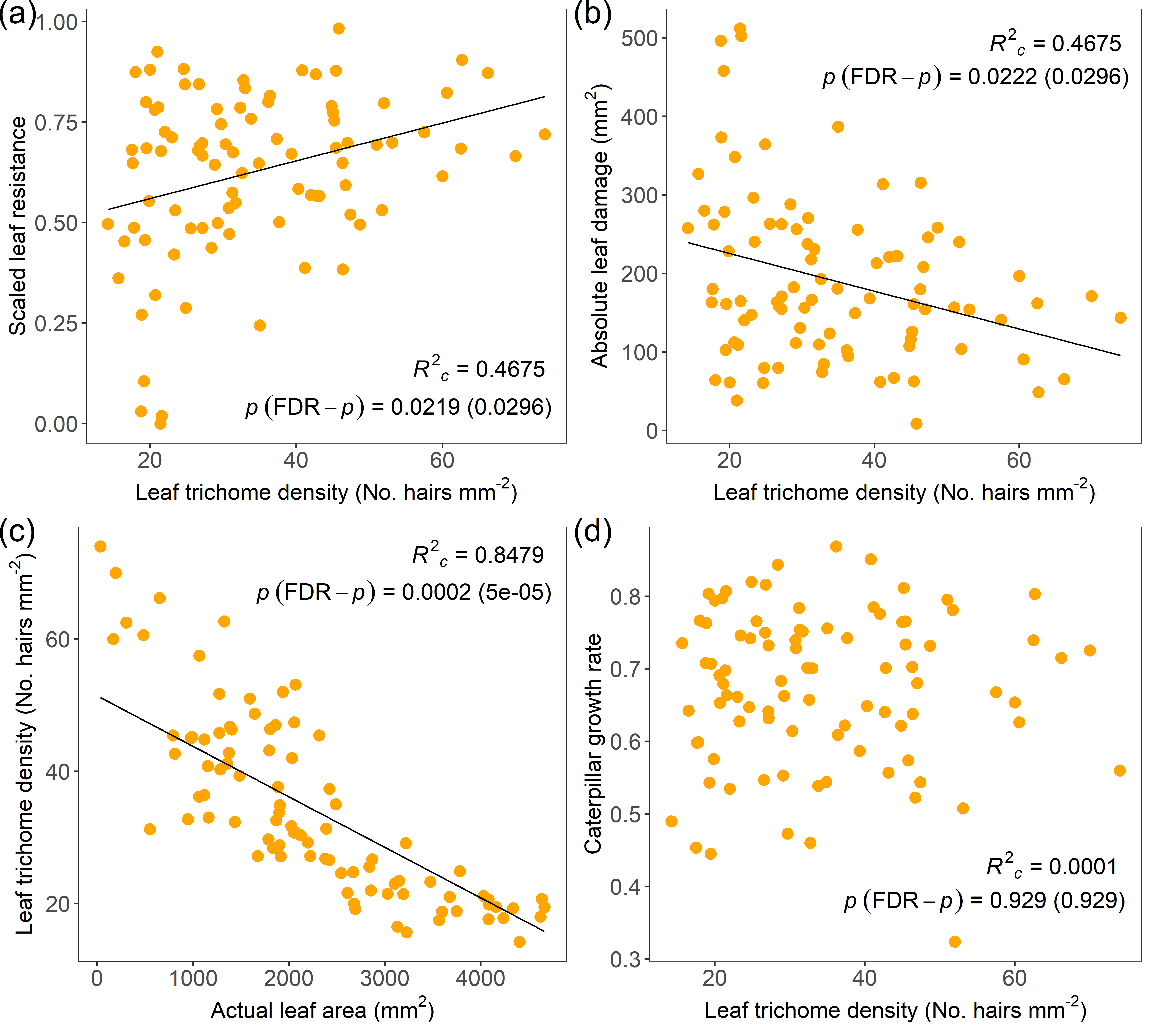


**Figure S12.** Results of linear mixed-effect models that test relationships between leaf trichome density and scaled leaf resistance, absolute leaf damage, leaf area of *A. thaliana* and caterpillar (*P. brassicae*) growth rate. Genotype was included as random effect in all four models. Fitted lines are drawn based on modelled intercepts and slopes. Model *R*^2^*_c_*, parametric bootstrap *p-*values and FDR-adjusted *p-*values are presented.


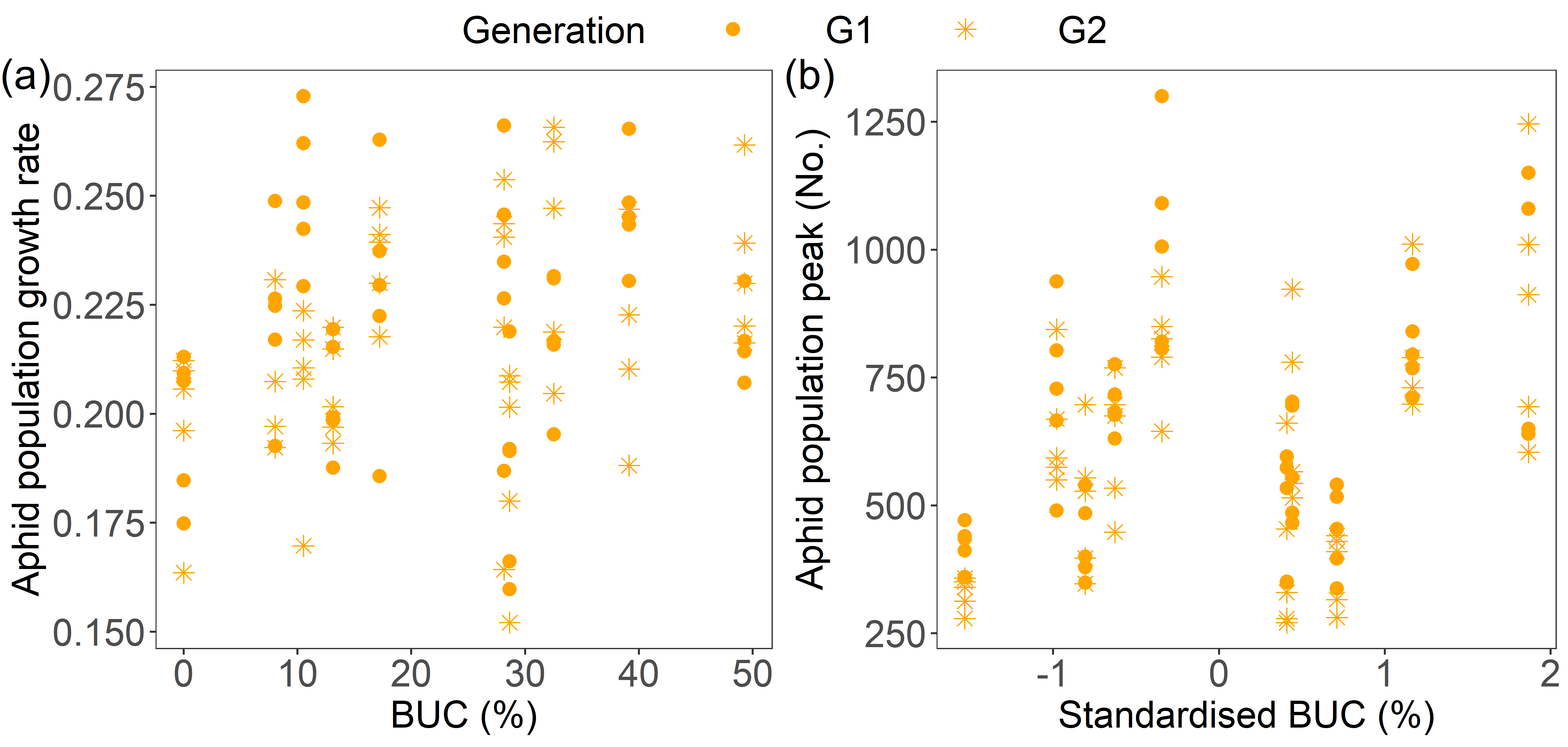


**Figure S13.** Aphid (*Myzus persicae*) performance as a function of urbanization (200 meter radius) and seed generation (G1, G2), as determined in a common garden experiment using 10 G1 and G2 genotypes of *Arabidopsis thaliana* whose mothers were located across an urban-rural gradient. Raw data are plotted against (a) percentage BUC and (b) standardized percentage BUC. For each variable, model *R*^2^*_c_*, estimates, parametric bootstrap *p* values and FDR-adjusted *p*-values of the fixed factors are given in Table S8.
